# The Role of Diet in Prognosis among Cancer Survivors: A Systematic Review and Meta-Analysis of Dietary Patterns and Diet Interventions

**DOI:** 10.3390/nu14020348

**Published:** 2022-01-14

**Authors:** Carlota Castro-Espin, Antonio Agudo

**Affiliations:** 1 Unit of Nutrition and Cancer, Catalan Institute of Oncology—ICO, L’Hospitalet de Llobregat, 08908 Barcelona, Spain; ccastro@idibell.cat; 2 Nutrition and Cancer Group, Epidemiology, Public Health, Cancer Prevention and Palliative Care Program, Bellvitge Biomedical Research Institute—IDIBELL, L’Hospitalet de Llobregat, 08908 Barcelona, Spain

**Keywords:** systematic review, meta-analysis, dietary pattern, prospective cohort, randomised controlled trial, cancer prognosis, cancer survival, dietary intervention

## Abstract

Cancer survival continues to improve in high-income countries, partly explained by advances in screening and treatment. Previous studies have mainly examined the relationship between individual dietary components and cancer prognosis in tumours with good therapeutic response (breast, colon and prostate cancers). The aim of this review is to assess qualitatively (and quantitatively where appropriate) the associations of dietary patterns and cancer prognosis from published prospective cohort studies, as well as the effect of diet interventions by means of randomised controlled trials (RCT). A systematic search was conducted in PubMed, and a total of 35 prospective cohort studies and 14 RCT published between 2011 and 2021 were selected. Better overall diet quality was associated with improved survival among breast and colorectal cancer survivors; adherence to the Mediterranean diet was associated to lower risk of mortality in colorectal and prostate cancer survivors. A meta-analysis using a random-effects model showed that higher versus lower diet quality was associated with a 23% reduction in overall mortality in breast cancer survivors. There was evidence that dietary interventions, generally combined with physical activity, improved overall quality of life, though most studies were in breast cancer survivors. Further cohort and intervention studies in other cancers are needed to make more specific recommendations.

## 1. Introduction

The term cancer survivor is generically applied to people living with a cancer diagnosis, including those who have been cured or recovered from the disease [1]. Although this definition includes people who have been diagnosed but have not yet started treatment, as well as patients being treated, and those who are at an advanced stage of the disease, in the present review we refer specifically to people who have been treated and have had a satisfactory response to treatment. For cancer survivors the main threat to their health in the short and medium term is the reappearance of the disease (recurrence), which can be local or distant (metastasis); the latter is, in turn, a strong determinant of survival. According to the most recent estimates, there were 44 million persons living with cancer in 2020 who had been diagnosed within the last 5 years [2]. That is, the high prevalence of cancer survivors is becoming a major health and social problem.

The diagnosis and treatment of cancer have experienced important advances in recent decades. Especially in the most developed countries, the practice of screening for breast cancer, and to a lesser extent for colon and rectal cancer, has spread. In addition, opportunistic screening for prostate cancer and some other tumours (thyroid, lung) is assiduously practiced. Furthermore, there have been substantial advances in the management and treatment of many tumours. As a result of these improvements, 5-year survival from colon, rectal and breast cancers has increased steadily in most developed countries for patients diagnosed during 2005–2009 [3]; survival for colon and rectal cancer reached 60% or more in 22 countries around the world, while for breast cancer, survival rose to 85% or higher in 17 countries worldwide. Striking increases in prostate cancer survival have occurred in many countries, reaching 95% in most developed countries, but trends vary widely.

Although the factors associated with higher or lower cancer incidence (risk or protective factors) do not necessarily must have prognostic value, it seems quite straightforward to think that determinants of the occurrence of a tumour may have some effect on the progression or recurrence of the disease, including the occurrence of a second tumour. Thus, the interest in the possible role of diet in cancer prognosis has been mostly focused on tumours for which diet is a widely recognised risk or protective factor. On the other hand, this area of research has been directed towards frequent tumours for which therapeutic alternatives with good response are available. Therefore, the results on the possible role of nutrition and related factors in the prognosis are concentrated mainly in breast, colon and prostate cancers [1].

Despite the apparent similarity or parallelism between the studies on the determinants of risk and prognosis, there are important differences in their research framework. First of all, the design option: although case-control studies are less and less used in nutritional epidemiology oriented to etiological research, in the case of prognostic determinants, where the outcome is often mortality, this option is not suitable. Only well-designed prospective cohorts are a suitable design for observational studies aimed to assess prognosis in this setting. On the other hand, intervention studies (i.e., randomised controlled trials, RCT) are needed and always preferred to establish the prognostic value of dietary factors with a high degree of evidence. The RCT are always complex and expensive; however, as they can be conducted in the clinical setting and the expected events are relatively common (at least compared with population studies looking for incidence), they should be, at least in theory, more prevalent than in etiological research. An additional problem has to do with the outcome, or rather, the variability in the possible outcomes. Indeed, while in the studies on risk factors the result is unique (diagnosis of an incident case of the disease), in the evaluation of the prognosis we can consider several outcomes: mortality (overall), death by a specific cause, recurrence, occurrence of a second tumour, a surrogate or marker of progression, or quality of life. Finally, there is the time frame of exposure (diet) assessment. Time-to-event analyses when the outcome is mortality (or recurrence) take the date at diagnosis as the entry time; therefore, ideally the dietary assessment should be as close to that date as possible. Two main time frames are considered when assessing prognosis: dietary factors collected pre- or post-diagnostic. Moreover, the time from dietary assessment to diagnosis, or conversely, from diagnosis to dietary assessment, must be considered. If this period is too long, it may call into question the validity of the study. Although there is not a clear consensus about this issue, most studies tend to restrict the dietary assessment to one year prior or after the date of diagnosis.

A comprehensive review [4] reported that physical activity after treatment may confer a number of health benefits to cancer patients, and that there is evidence to suggest that elevated body fatness is a predictor of poor outcome in breast cancer survivors. With regard to diet, this review reported that there is evidence of links between better survival after breast cancer and eating foods containing fibre, soya, and lower intakes of total and saturated fats. However, due to limitations of much of the existing research, the evidence is not strong enough to make specific recommendations. Several reviews summarising the observational evidence from prospective cohorts of cancer survivors have been published in the last ten years [5,6,7]. All of them reported associations between mortality and some foods or groups of foods among survivors of several common cancers. On the other hand, a recent review of the quality of five evidence-based nutrition guidelines for cancer survivors [8] reported that limited information on nutrition was available in these guidelines, with the focus being on the promotion of fruit, vegetables and wholegrains and reducing fat, red meat and alcohol. There was also a tendency to recommend cancer prevention guidelines be used for cancer survivors rather than developing specific guidance for this group.

A couple of issues about the major conclusions of these reviews are worth considering. First, as already noted, most of the observational evidence summarised concerns individual foods, food groups or single nutrients. However, food consumption cannot be considered in isolation, but in combination with others. Therefore, examination of the survivor’s diet as a whole, by means of dietary patterns, could be more readily translated into dietary guidelines. This seems particularly relevant for assessing protective effects: while there are several examples of dietary components that can increase the risk of cancer (e.g., alcohol) there are few (if any) examples of single nutrients or components that directly decrease cancer risk [9]. This can translate into disease progression, risk of recurrence or death. By means of dietary patterns assessment, studies may try to look at the whole diet, which is likely to have interactive, synergistic and combined effects on disease risk and progression [10].

On the other hand, in reviews discussed above [4,8], a claim was made that further research, mainly from intervention studies, is needed to make specific recommendations for cancer survivors. In fact, it is not entirely true that clinical trials on the effect of diet as a prognostic factor in cancer survivors are lacking: during the first decade of this century, results of two large RCT evaluating the effect of dietary intervention on the risk of recurrence of breast cancer were published [11,12]. However, they did not provide clear support for a role of diet owing to their discrepant results. The Women’s Intervention Nutrition Study (WINS) [11] assigned 2437 women with early stage breast cancer to either a low-fat or standard diet. After approximately five years of follow up the intervention group had a significant 24% lower risk of recurrence compared to the control group. In contrast, the Women’s Healthy Eating and Living Study (WHEL) [12], including 3088 breast cancer patients, found that an intervention diet rich in vegetables, fruit and fibre, and low in fat compared to a control diet did not reduce risk of recurrence or mortality after a 7-year follow up. Several reasons have been put forward to explain these discrepancies; however, the most remarkable difference is that in WHEL there was no significant weight modification in either the control or intervention group, whereas in WINS there was a significant, though unplanned, weight reduction in the intervention arm [13]. These results suggest that energy balance may play a significant role in breast cancer prognosis and may be more important than the modest effects of reducing total fat intake or modifying other dietary factors. The growing evidence suggesting the relevant role of weight control on breast cancer recurrence, together with evidence of the beneficial effects of physical activity among cancer patients [14,15], led to the development of lifestyle interventions combining dietary and physical activity components as the best strategy to improve prognosis and quality of life among survivors of breast and other cancers.

Keeping in mind the issues discussed above, the aim of this study was to conduct a systematic review and meta-analysis of prospective cohort studies and randomised controlled trials that investigated the effects of dietary patterns and dietary interventions on the prognosis among cancer survivors. We adopted a broad definition of prognosis, including all the events and outcomes with prognostic significance: overall and cancer-specific mortality, recurrence, markers of disease progression and quality of life.

## 2. Materials and Methods

This systematic review adhered to the Preferred Reporting Items for Systematic Reviews and Meta-Analyses (PRISMA) statement [16] and followed a pre-planned unpublished protocol that can be requested by contacting the corresponding author.

### 2.1. Search Strategy

The authors conducted a total of seven literature searches using combinations of several keywords related to diet and cancer prognosis in PubMed database, from 1 January 2011 until 31 August 2021. No restriction on language was made and only peer reviewed sources limited to human adult studies were included. When articles were reviews and/or meta-analyses only those published on the previous five years were included to further explore other relevant references. The following search strategy was used: (cancer OR neoplasm) AND (dietary pattern OR food-stuff OR food nutrients OR diet) AND (mortality OR prognosis OR cancer mortality OR cancer survival OR cancer prognosis OR cancer outcomes OR cancer recurrence OR cancer survivors) AND intervention. Further exploration of the reference lists of the identified papers complemented these searches. Any disagreement was resolved through discussion between the two authors.

### 2.2. Study Selection 

**Figure 1:**
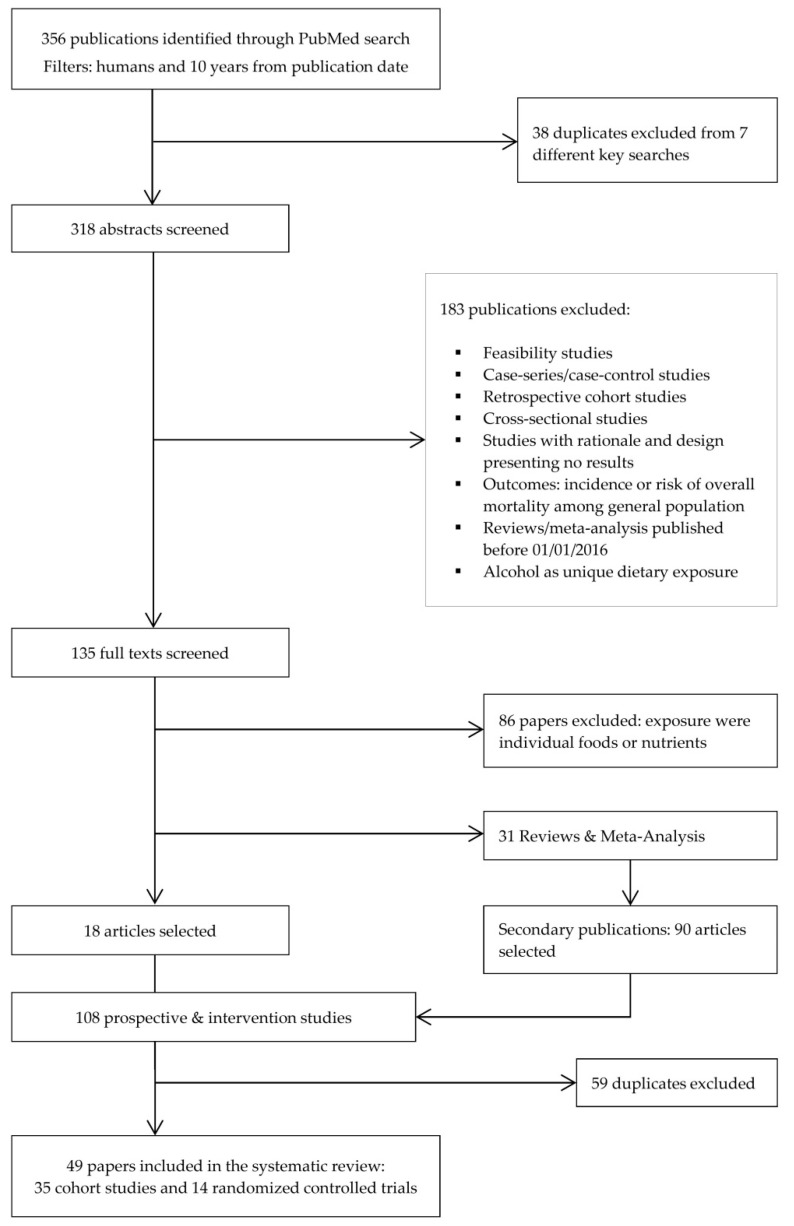
Flow diagram of literature search and selection process adapted from Preferred Reporting Items for Systematic Reviews and Meta-Analyses (PRISMA).

The authors reviewed the titles and abstracts of all articles and selected studies that met the following criteria: (1) prospective cohort or randomised controlled trial (RCT) design; (2) available in full-text; and (3) assessing the relationship between dietary patterns (in cohorts) or dietary intervention (in trials) and prognostic-related outcomes (i.e., all-cause mortality, cancer-specific mortality, recurrence and quality of life (QoL)). For RCT, studies including dietary interventions either alone or in combination with physical activity were considered. We excluded feasibility, cross-sectional, case-series or case-control studies, retrospective cohorts, studies focused on the rationale and design presenting no results, any study whose population is not clearly defined as cancer survivors, as well as reviews or meta-analysis published before 1 January 2016 and exposure considering only alcohol (Figure 1).

### 2.3. Data Extraction

The following information was extracted from each selected study: reference (author, year), country, population details (clinical features, sample size, age, and follow-up time of the cohort or trial), dietary assessment tool with its main relevant features, outcomes, results, and observations (e.g., adjustment for confounders). For the RCT we included a description of the intervention and the methods used for the assessment of quality of life, as many of them investigated this outcome. Where multivariable models were reported, the model including the set of potential confounders judged as the most adequate was selected. 

### 2.4. Outcomes

The primary outcome usually was of time-to-event type. Survival was mostly measured as overall or cancer-specific mortality, as well as disease-free survival (or risk of recurrence). Other selected outcomes related to prognosis were different dimensions of quality of life.

### 2.5. Bias Assessment

The risk of bias was assessed by means of the Newcastle-Ottawa Scale (NOS) for cohort studies [17]. The NOS contains eight items, categorised into three dimensions including selection, comparability and outcome (Table A1). For each item a series of response options is provided, and a star system is used, whereby the highest quality studies are awarded with a maximum of one star for each item with the exception of the item related to comparability, which allows the assignment of two stars. Therefore, the NOS score ranges from zero to nine.

### 2.6. Meta-Analysis

Eligible studies for meta-analysis were those that studied the same outcome, same exposure and same cancer type; a meta-analysis was performed only for sets of three or more studies that fulfilled the above-mentioned criteria. According to this, we conducted a meta-analysis of four cohort studies on breast cancer survivors, looking at overall and specific mortality in relation to dietary patterns reflecting diet quality [18,19,20,21].

We used the adjusted hazard ratio (HR) as an estimate of the relative risk of each study to calculate a summary effect estimate applying two different approaches. First, we used estimates for the fourth quartile [19,20,21] or the fifth quintile [18] as compared with the reference (first quartile or quintile) to calculate the effect of the highest versus the lowest level of the diet quality index. On the other hand, we calculated an estimate of the effect (with its corresponding 95% confidence interval) associated with each 10-unit increase of the index using the mean or the midpoint of each category, by means of a method based upon generalised least squares [22]. The overall HRs were estimated by means of a random effect model [23,24]. Heterogeneity across studies was assessed by means of the I^2^ statistic [25], together with a prediction interval [26]. All the data used to perform the meta-analysis can be found in Table A2.

## 3. Results

### 3.1. Identified Studies

From the initial search, 356 records were identified (Figure 1) of which 318 were selected for title and abstract screen after removing duplicates. Of these, 183 were excluded, leaving 135 full-text articles for review. Additionally, prospective cohort studies where exposure was a single food, nutrient or food group were excluded, leaving 18 articles. Moreover, 90 new articles were identified through the systematic screening of references in reviews and meta-analyses found in the previous step, resulting in 108 articles selected. After removing duplicates, 49 papers in total were ultimately retained for the present review.

### 3.2. Prospective Cohort Studies

A total of 35 prospective cohorts were identified. Details of these studies are shown in Table 1. The majority of studies were conducted in North America (26 in the US, including one that combined data from Mexico, and two from Canada); four were conducted in Europe, two in Asia, and one in Australia. Most cohorts included breast and colorectal cancer survivors (13 and 11 studies respectively), followed by three studies of survivors of prostate cancer, two studies of head and neck cancers, two studies of ovarian cancer, and one study each of bladder cancer and multiple myeloma. The two remaining studies included survivors of a combination of several tumours.

All but six studies used a food frequency questionnaire (FFQ) to assess diet intake. Ten studies collected dietary data before diagnosis, twenty after diagnosis and five assessed diet both before and after diagnosis. Six studies built a diet pattern by means of statistically derived methods (i.e., Prudent/Western pattern; Healthy/Unhealthy pattern); most of the remaining studies (*n* = 26) used *a priori* defined indices, for example, based on dietary guidelines (i.e., Healthy Eating Index [HEI]-2005; Alternative Healthy Eating Index [AHEI]-2010; Mediterranean Diet Score [MDS]), and three studies included both approaches.

Overall, the cohort studies had a good quality as measured by the NOS Quality Assessment Scale (Table A1), with an average score of 7.8 (scale with range 0–9). Seven studies graded the maximum 9 points of the scale, seventeen graded 8 points, seven graded 7 points and the remaining three graded 6 or 5 points.

#### 3.2.1. Breast Cancer (BC)

Five out of thirteen prospective studies focused on postmenopausal BC patients and eleven studies included overall mortality and breast cancer-specific mortality as outcomes. Other outcomes of interest were recurrences [18,27,28,29] and breast cancer-specific events, defined as recurrence or metastasis of breast cancer and breast cancer deaths, which was only reported in one study [21].

A total of seven studies assessed diet using the HEI or AHEI indices. The HEI is a measure of diet quality in relation to the Dietary Guidelines for Americans (DGA) with different versions updated over the years; the AHEI captures evidence-based recommendations that incorporate additional food- and nutrient-focused components to predict chronic disease risk [30]. For instance, the DGA 2015 has moved in the direction of the AHEI and the HEI-2015 has included new components present in the AHEI. The different versions of HEI and the AHEI-2010 are similar in several aspects. 

A study based on the Nurses’ Health Study (NHS) [18] found no association between four different diet quality indices, including the AHEI, and breast cancer survival among postmenopausal survivors. The same cohort examined the association with AHEI for all survivors with an extended follow-up, and only found a significant reduced risk (43%) of non-breast cancer-related mortality [28]. In contrast, the remaining two studies that assessed different versions of the HEI index, reported significant lower risk for all-cause mortality with higher adherence to the score [19,31] though the smaller sample size. When restricted to postmenopausal women, the Women’s Health Initiative (WHI) study also observed a reduction in risk (36%) of all-cause and (42%) non-breast cancer-related mortality according to greater HEI-2005 scores [20]. Updated versions of the HEI score in more recent publications showed an increased risk (66%) of breast cancer mortality for women who decreased their diet quality compared to women with stable diet quality [32], however increased adherence to the HEI-2015 in a large Chinese cohort showed no significant association with breast cancer mortality.

For the two studies that assessed the DASH diet in relation to breast cancer survival, only one reported a significant protective effect (34% reduction) against all-cause mortality and breast cancer-specific events (40% reduction), although the cohort included survivors with I to IV stages [21]. By contrast, previous findings in the NHS only observed a significant protective effect for non-breast cancer-related mortality [28]. 

Two different cohorts assessed the inflammatory potential of the diet. One cohort based in Korea found that greater adherence to a more inflammatory diet as measured by the Dietary Inflammatory Index (DII) was associated with an increased risk of recurrence and all-cause mortality [29]. In the same direction, restricted to postmenopausal survivors in a larger US cohort, adherence to a more anti-inflammatory diet was associated with a protective effect (66% reduction) against all-cause mortality [33]. 

The Diabetes Reduction Risk Diet (DRRD), which comprises 9 dietary components associated with 40% lower type II diabetes risk, showed a significant reduced BC-specific mortality (20%) and all-cause mortality (34%) comparing highest versus lowest quintile of adherence from a large US cohort study [34]. Conversely, two different versions of the Mediterranean Diet Score were found to be not significantly associated with all-cause mortality [18,31].

Among data-driven dietary patterns, only the ‘Unhealthy’ pattern assessed before diagnosis was associated with an increased risk of non-breast cancer-related mortality among postmenopausal women [27]. This study included survivors with advanced (stage IV) tumours; furthermore, multivariable models were not adjusted for body mass index and physical activity.

For scores based on dietary guidelines for health across different populations, adherence scores to the Chinese Food Pagoda (CHFP) in a large Chinese cohort showed decreased risk of all-cause mortality (34%) according to the CHFP-2007 version and a 33–36% reduced risk of breast cancer-specific events (i.e., recurrence, metastasis, or death related to breast cancer) according to CHFP-2007 and CHFP-2016 [21]. Conversely, dietary scores based on the American Cancer Society (ACS) recommendations were not significantly associated with better breast cancer survival [35] but scores that underline the WCRF/AICR guidelines showed a significant lower risk (39%) of all-cause mortality among breast cancer survivors [36]. 

##### Meta-Analysis of Cohort Studies on Breast Cancer Survivors

Candidate studies for meta-analysis were those assessing common outcomes (i.e., all-cause mortality and breast cancer-specific mortality) in relation to a dietary pattern reflecting the quality of diet. The diet quality indices selected were the HEI-2005 [19,20], the HEI-2015 [21], and the AHEI [18]. They have a common background, are close to each other and are similarly associated with chronic disease risk [37]. All of them have a scale from 0 to100.

**Figure 2:**
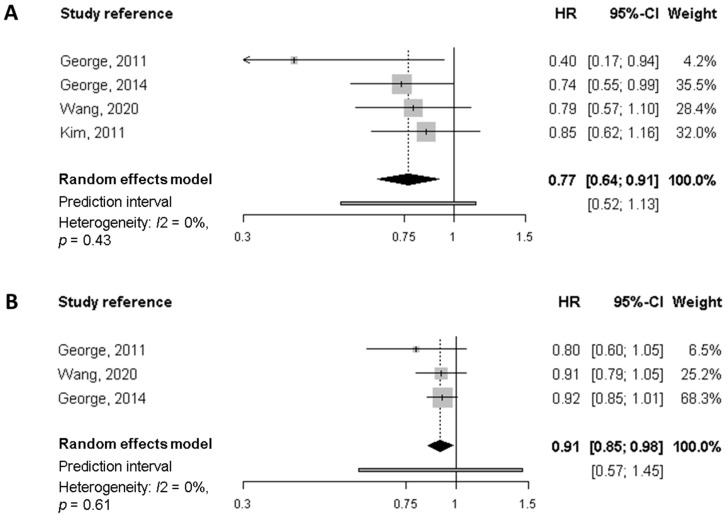
Meta-analysis of prospective cohort studies on the association between quality diet score and overall mortality among breast cancer survivors. (**A**) Forest plot showing pooled hazard ratios (HRs) with 95% CI for the highest diet quality (Healthy Eating Index [HEI], Alternate Health Eating Index [AHEI]) vs. lowest diet quality category for overall mortality. (**B**) Forest plot showing pooled HRs with 95% CI for 10-point increase in the quality diet score and overall mortality.

**Figure 3:**
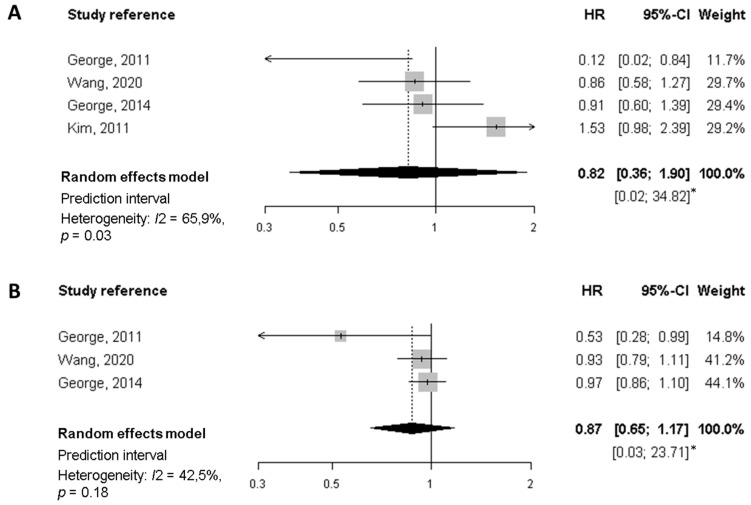
Meta-analysis of prospective cohort studies on the association between quality diet score and breast cancer-specific mortality among breast cancer survivors. (**A**) Forest plot showing pooled hazard ratios (HRs) with 95% CI for the highest diet quality (Healthy Eating Index [HEI], Alternate Health Eating Index [AHEI]) vs lowest diet quality category for breast cancer-specific mortality. (**B**) Forest plot showing pooled HRs with 95% CI for 10-point increase in the quality diet score and breast cancer-specific mortality. * Prediction interval lines are not represented in this figure because intervals are too wide.

Regarding all-cause mortality, the summary HR of the highest quality diet versus the lowest was 0.77 (95% CI, 0.64 to 0.91), based on estimates from four studies (Figure 2). Similarly, each 10-points increase in the score (increasing overall diet quality), which is equivalent to a jump from one quartile to the next, was associated with a significant 9% reduction of mortality (HR 0.91, 95% CI, 0.85 to 0.98). In neither case was there evidence of heterogeneity. For breast cancer-specific mortality (Figure 3) the summary HR was 0.82 (95% CI, 0.36 to 1.90) when comparing the highest versus lowest categories of diet quality, whereas no significant decrease in BC-mortality was found for each 10-point increase in the score. Potential heterogeneity was present (*I*^2^ = 66%, *p* = 0.03) for the highest versus lowest diet quality score. This is also reflected in the wide prediction interval, which indicates the uncertainty we could expect in the summary effect if a new study is included. Indeed, a meta-analysis with few studies is usually expected to report an imprecise prediction interval [38].

#### 3.2.2. Colorectal Cancer (CRC)

Most of the eleven studies selected used *a priori* dietary indices based on literature or derived from guidelines (e.g., WCRF/AICR guidelines, HEI score) to assess overall dietary intake. Only three studies, two from Canada [39,40] and one from the US [41], examined data-driven dietary patterns. A higher adherence to the pre-diagnosis ‘processed meat pattern’, characterised by a high intake of processed meat, red meat, fish and processed fish, was associated with worse disease-free survival (defined as first occurrence of death, recurrence or metastasis) among all CRC survivors, especially for colon cancers, and with an increased risk of overall mortality in colon cancer survivors [39]. Further analyses in the same cohort [40] found that clusters characterised by high intake of meat and dairy products and high intake of refined grains, sugar and soft drinks, compared with a reference cluster characterised by higher intake of fruits, vegetables, whole grains and wine, showed poorer survival (higher risk of mortality, recurrence and metastasis). On the other hand, a pattern high in refined grains and sugar/soft drinks was also associated with an increased risk of overall mortality. In contrast, the ‘Prudent’ (healthy) and ‘Western’ (unhealthy) patterns were not associated with the overall or CRC-specific mortality in women in a different study [41]. 

The most common *a priori* pattern used to study the overall and CRC-specific mortality was the Mediterranean Diet, present in a total of four studies. For pre-diagnosis assessments, lower adherence to the Alternate Mediterranean Diet Score (altMED) was significantly associated with 62% increase in overall mortality [40]. In addition, results from the large Multiethnic Cohort study (MEC) also reported a protective effect when moving from lower adherence to higher in the score for both CRC and all-cause deaths but limited to African-American women [42]. Similarly, in post-diagnosis assessment in a large German cohort, a lower overall mortality risk was found among men and women comparing extreme quartiles for higher adherence to the Modified Mediterranean Diet (MDD) score (adapted to non-Mediterranean countries) and also for a 1-point increase in score [43]. These findings, however, were not supported by results in other large cohort and no association was found for overall or specific mortality in women survivors of CRC [41].

Higher compared to lower adherence to the HEI-2005 dietary pattern before diagnosis showed a significant protective effect both for CRC-specific and overall mortality (36 and 40% reduction respectively, limited to rectal cancer survivors) [44]. Conversely, results from the MEC study found no association when all CRC survivors were analysed [42]. Among women CRC survivors from the NHS, a significant inverse association was found between the highest versus lowest quintiles of the AHEI-2010 assessed after diagnosis and overall mortality [41].

Two studies reported no association between the DASH diet and overall and specific CRC mortality [41,42]. On the other hand, higher adherence to the Healthy Nordic Food Index (HNFI) was inversely associated with all-cause mortality (37% reduction as compared to lower adherence) and a significant 10% reduction for each 1-point increase in the score [43]. 

A Canadian study [40] examined the association between the inflammatory potential of diet after diagnosis and all-cause and specific mortality, but no association was found. However, the WHI cohort, including only women, using a modified version of the same index (E-DII) taking into account diet plus supplements intake, reported a lower all-cause mortality for those following the most anti-inflammatory diets (51% significant reduction compared to the most pro-inflammatory diets) [45].

Another study conducted within the NHS and Health Professionals Follow-up Study (HPFS) cohorts revealed that higher adherence to the empirical dietary index for hyperinsulinaemia (EDIH) had a 66% increased risk of dying from CRC and a 24% increased risk of death from all causes [46].

Finally, results from the European Prospective Investigation into Cancer and Nutrition (EPIC) study indicated that higher concordance with the WCRF/AICR recommendations on diet, physical activity and body fatness prior CRC diagnosis was associated with improved overall and specific survival among CRC patients [47]. A previous study with a much smaller number of survivors who were asked to follow the same recommendations after diagnosis reported non-significant results [36]. It is worth mentioning, however, that this study did not report details of cancer stage of participants and did not include specific adjustment for lifestyle confounders.

#### 3.2.3. Other Cancers

This section includes studies that examine several types of cancers together, as well as studies dealing with survivors of cancers of the prostate, head and neck, ovary, urinary bladder and multiple myeloma. 

Two studies included several cancers, both conducted in two large cohorts of women [31,36]. The first one, from the Iowa Women’s Health Study (IWHS), examined adherence to the WCRF/AICR guidelines among older women survivors of breast cancer, colorectal cancer, gynaecologic cancers (including cervical, endometrial, ovarian and related cancers) and other cancers [36]. The results showed that women with the highest versus the lowest adherence to guidelines of WCRF/AICR after diagnosis had a significantly better overall survival. The second analysed the HEI and Mediterranean Diet scores on the following gynaecological cancers: ovarian, cervical and uterine cancer [31]. Of the two dietary patterns assessed, only the HEI score was significantly associated with all-cause mortality, both for each unit increase in the score and also comparing good versus poor adherence.

Three cohorts examined different dietary patterns in relation to prostate cancer prognosis, two based in the US [48,49] and one in Italy [50]. All but one [50] accounted for key variables of adjustment (obesity, physical activity, alcohol consumption and smoking habit). Only one of the three studies used data-driven dietary patterns and found that higher adherence to a ‘Western’ dietary pattern was borderline associated with higher prostate-specific mortality and significantly associated with all-cause mortality, while a ‘Prudent’ dietary pattern was significantly related to lower all-cause mortality [49]. In a large cohort of prostate cancer survivors a higher adherence to a Mediterranean diet score was significantly associated with a 22% lower risk of overall survival [48]. On the other hand, a strong (and significant) relationship was observed in patients with Gleason 7–10 (more aggressive, poor-prognosis cancers) following more pro-inflammatory diets for prostate cancer-specific mortality [50].

Two studies on head and neck cancers survivors from the US used pre-treatment data-derived dietary patterns [51,52]. There was a significant inverse association between better adherence to a ‘whole-foods’ pattern (characterised by high intakes of vegetables, fruit, fish, poultry and whole grains) and a decrease (44%) in overall mortality [51]. The second study, which examined the nutrition impact symptoms burden among head and neck cancer survivors, reported that a ‘Prudent’ pattern was significantly associated with a reduction in these symptoms (i.e., difficulty chewing, dysphagia of liquids and solid foods, and mucositis) [52]. The assessment of potential confounders was incomplete and inconsistent in both studies.

For ovarian cancer, two studies assessed the effect of different diet patterns in relation to cancer survival. In a study based in the US [53], survivors with a higher quality diet prior to diagnosis according to the HEI-2005 score presented a significantly lower risk (27%) of all-cause mortality, not significant for ovarian cancer-specific mortality. On the other hand, in a study conducted in Australia [54], the Healthy Lifestyle Index (HLI) (that included smoking status, height, weight, physical activity, diet quality score and alcohol) after diagnosis was inversely associated with lower overall mortality; however, when its components were analysed individually, a higher adherence to the diet quality score (defined and quantified using the WCRF/AICR score) was not associated with overall better survival.

Finally, a significant association was observed between the data-driven ‘Western’ pattern and risk of recurrence (48% increased risk) compared to the lowest adherence for urinary bladder cancer survivors [55]. Similarly for multiple myeloma survivors, a study within the NHS and HPFS cohorts found that the ‘Western’ dietary pattern was significantly associated with an increased risk of specific and overall mortality. In addition, survivors with healthier pre-diagnosis dietary patterns, specifically AHEI-2010, aMED, DASH and the ‘Prudent’ pattern, reported better overall and specific survival [56].

### 3.3. Randomised Controlled Trials (RCT)

A total of fourteen RCT were identified; the details and main features of these studies are shown in Table 2. Clinical trials were mostly from Europe (six studies) and the US (six studies, including a RCT conducted in the US and Canada); the remaining two RCT were carried out in Asia (South Korea and China). Eight studies focused on breast cancer survivors, three on colorectal cancers (including one exclusively on colon cancer), one on prostate cancer survivors, one study on endometrial cancer survivors and finally one study targeted survivors from several cancer subtypes (i.e., breast, stomach, colon, and lung cancer). Three of the fourteen studies were randomised controlled pilot trials [57,58,59] and hence included a small number of participants. The remaining RCT included a number of participants on the order of a few hundred, with a range from 38 to 3374. The primary outcome of three RCT was survival or cancer progression, but the most common outcomes were quality of life dimensions (i.e., fatigue, sleep quality, physical and mental function).

#### 3.3.1. Randomised Controlled Trials on Breast Cancer Survivors

Two out of the eight RCT included only breast cancer survivors who were overweight or obese at start of the intervention [60,61] and one study focused exclusively on triple-negative BC survivors [62]. A total of four studies included interventions combining nutritional counselling and physical activity programme, targeting participants in the intervention groups generally with the primary goal of reducing energy intake [63,64], dietary fat [62] or weight change [65]. All but two studies [60,66] had as primary or secondary outcomes changes in quality of life assessed by means of different questionnaires (i.e., Function After Cancer Therapy [FACT], European Organization for Research and Treatment of Cancer Quality of Life Questionnaire [EORTC QLQ-C30], Short Form Health Survey [SF-36]). Some RCT defined outcomes as changes in the lifestyle components of the intervention (i.e., foods, groups of foods or nutrients, physical activity) or intervention-related parameters (i.e., weight, body mass index). We did not take into account these outcomes in our review as they do not have a clear prognostic meaning or cannot be considered as surrogates or prognosis.

Quality of life, as measured by the FACT-B (specific scale for breast cancer), showed significant improvements in the intervention group for survivors that followed a 6-month individualised exercise and a hypocaloric healthy eating programme [63]. Similarly, a shorter intervention that combined moderate physical activity and nutrition advice with the goal to decrease dietary fat by 200 kcal weekly, improved quality of life (measured by the FACT-B total score) among triple-negative BC survivors [62]. In addition, mean change in EORTC QLQ-C30 physical condition score was significantly greater for women in the telephone-based weight loss intervention (versus the mail-based arm) among postmenopausal BC survivors in the LISA study [60]. Actually, the LISA study had disease-free survival and overall survival as primary outcomes, but the results were not reported due to lack of financial support to reach the sample size initially planned. In a large study, the ENERGY trial (344 participants in intervention arm, 328 in the control arm) reported a weak or null associations after a 24-months intervention assessing specific items of the SF-36 with a nutritional weight loss programme among breast cancer survivors with overweight or obesity (BMI > 25) [61]. 

A unique 2-week intervention in hydrothermal centres that included physical activity and nutritional education with calorie restriction (1200 kcal/day) reported improvements on breast cancer patients’ quality of life according to the SF-36 global score at several times of follow-up, with the highest difference between group arms at 6 months [64]. In a randomised pilot trial investigating the effect of a 3-month ‘fatigue reduction diet’ (defined as a diet rich in fruit, vegetables, whole grains, and foods rich in omega-3 fatty acids) revealed an improvement in fatigue and sleep quality in 15 breast cancer survivors compared to the15 participants from the control group. In contrast, in a RCT with 72 cases (36 in each study group) there was no significant change in global health and fatigue with a 6-month intervention including dietary counselling and physical activity sessions, although only half of the participants in the intervention group completed at least 75% of the programme sessions [65].

Finally, the Women’s Health Initiative (WHI) Dietary Modification (DM) clinical trial, with a long dietary intervention (8.5 years) and extended follow-up (median 19.6 years), reported that the adoption of a low-fat dietary pattern (characterised by increased vegetable, fruit and grain intake) reduced significantly the risk of overall (15%) and breast cancer-specific mortality (21%) among postmenopausal women [66].

#### 3.3.2. Randomised Controlled Trials on Other Cancers

The Leadership and Coaching for Health (LEACH) program, a 12-month intervention based on counselling for balanced dietary habits, physical activity and distress management, improved anxiety according to the Hospital Anxiety and Depression Scale (HADS), social functioning and appetite loss scores from baseline to 3 months in survivors of several tumour sites (breast, stomach, colon, lung) with favourable prognosis (non-metastatic cases with treatment completed within the last two years). In addition, from baseline to 12 months, the intervention group showed a significantly greater decrease in the EORTC QLQ-C30 (European Organization for Research and Treatment of Cancer Quality of Life Questionnaire) fatigue score [67].

Three RCT were conducted on CRC survivors, including a small (18 participants, 9 per arm) randomised pilot study [58]. An improvement on fatigue score after a 12-week program of home-based exercise and dietary advice in the intervention versus control group was reported, but no change in cancer-specific quality of life according to the FACT-C (Function After Cancer Therapy-Colorectal) was observed. A recent study in China reported that participants receiving a 12-month dietary intervention (aimed to reduce red/processed meat to less than 5 servings/week [with processed meat less than 2] and limiting refined grains to 2 servings/day) experienced a significant improvement in generic and CRC-specific QoL, and reduced levels of depression at 12 and 24 month of follow-up [68]. On the other hand, the double-blind, phase III, randomised, placebo-controlled trial providing daily antioxidant supplementation (active compound of 200 μg selenium, 30 mg zinc, 2 mg vitamin A, 180 mg vitamin C, 30 mg vitamin E) for 5 years reported a significant 39% reduction of recurrence risk in the intervention compared to the placebo group in CRC patients post-polypectomy [69].

Regarding prostate cancer survivors, there were no significant differences in time to progression for participants of the MEAL (Men’s Eating and Living) study that received a telephone-counselling intervention addressed to increase vegetables consumption over a 24-month period compared to the control group, which received written information on diet and prostate cancer [70]. 

Finally, in a randomised pilot study in endometrial cancer survivors, an 8-week intervention based on healthy eating and physical activity sessions was associated with a significant improvement in global quality of life (as measured by the EORTC QLQ-C30) in the intervention arm at 24 weeks compared to the control group [59].

## 4. Discussion

This systematic review summarises the evidence of the impact of diet, as measured by dietary patterns and nutritional interventions, on cancer prognosis, based upon thirty-five prospective cohort studies and fourteen randomised controlled trials. As expected, the vast majority of the articles focused on breast and colorectal cancer survivors.

A better overall diet (i.e., with a high diet quality index) may improve survival after breast cancer diagnosis. The evidence is rather limited to draw conclusions about breast cancer specific-mortality and recurrence. A meta-analysis of four prospective cohort studies including over 9200 breast cancer survivors estimated that women in the highest versus the lowest category of diet quality index had a significant 23% lower mortality. Moreover, for a 10-point increase in the score, which is equivalent to moving from one quartile to the next, there was a significant 9% reduction in mortality. Although the point estimates were similar for breast cancer-specific survival, the association with a better diet quality turned out to be non-significant.

We identified evidence of an increased risk of overall mortality for breast cancer survivors following more pro-inflammatory diets [29]. However, the effect of the inflammatory potential of diet on breast cancer progression needs to be confirmed in larger studies. In fact, these findings are in good agreement with previous studies showing an association between better post-diagnosis diet quality and lower levels of chronic inflammation, as measured by C-reactive protein, independent of body mass index or physical activity [71].

A wide variety of dietary patterns have been assessed for their prognostic value in colorectal cancer survivors. A potential protective effect for overall mortality was identified with Mediterranean dietary pattern, although the results need to be confirmed in other large cohorts and trials [43]. In contrast, the DASH diet (a dietary pattern in principle intended to reduce hypertension) revealed no association with colorectal cancer survival, based on results from two large cohorts [41,42].

The ‘processed meat’ pattern and two other clusters, the first characterised by meat and dairy intake, and another one characterised by intake of total grains, sugar and soft drinks, were associated with worse overall prognosis (combined mortality, recurrence, or metastasis) [40]. Instead, other derived patterns, the ‘Prudent’ and the ‘Western’ dietary patterns showed no associations with mortality outcomes in a different study [41]. The finding of a potential role in disease progression for processed meat is in good agreement with previous evidence confirming its role as a cause of colorectal cancer [72].

A better post-diagnostic diet quality, assessed by the HEI, was associated with lower mortality among female breast and gynaecological cancers [31]. A potential mechanism explaining these findings could be mediated through inflammation since higher quality diets after diagnosis exhibited lower C-reactive protein levels in cancer patients [73] and diets corresponding to higher adherence to HEI score are considered diets with low inflammatory potential [71]. Moreover, a higher adherence to the WCRF/AICR guidelines showed a better overall survival among older female cancers [36].

There seems not be enough evidence to draw conclusions on the prognosis of cancers other than breast and colorectal cancer, but according to three studies in prostate cancer survivors, a ‘Western’ dietary pattern and a diet with higher inflammatory potential were associated with higher overall and cancer-specific mortality, respectively [49,50]. In contrast, the Mediterranean diet, which is attributed with an anti-inflammatory potential, was associated with lower overall mortality [48].

The randomised clinical trials included in this review evaluated the effect of a dietary intervention, often in combination with physical activity, on cancer prognosis. Despite most of the studies focused on quality of life as primary or secondary outcome, differences in study design and tools used for QoL assessment did not allow us to calculate an overall estimate for each specific cancer. Three studies on breast cancer survivors reported significant improvement in quality of life following interventions aimed at weight loss or energy reduction, combined with physical activity advice [60,62,63]. However, a large study in overweight or obese patients reported no effect on quality of life after a long 24-month nutritional weight loss program [61]. Inconsistent results were found between two small trials on breast cancer survivors investigating fatigue, which is one of the most researched aspects of quality of life among cancer survivors; one was a pilot study, randomised and controlled, that reported improvement on fatigue after a 3-month diet rich in fruit, vegetables, whole grains and foods rich in omega-3 fatty acids [57], and the other did not see changes in fatigue after a 6-month intervention based on dietary counselling and physical activity sessions [65]. Key differences in the design of the studies may partly explain inconsistencies in results when examining the same outcome in the same type of cancer.

As for other cancers, generally, interventions that combined dietary counselling and physical activity improved overall quality of life among survivors, although evidence was limited to draw precise conclusions or make recommendations. 

### Study Strengths and Limitations

Strengths of this systematic review are the inclusion of dietary patterns instead of individual foods, food groups or nutrients as well as the restricted inclusion of only prospective cohort studies and randomised controlled trials. Furthermore, probably because of strict application of the selection criteria, the studies included in the review had good validity, according to the high score achieved on a scale designed to assess the risk of bias. In addition, examining the diet as a whole provides a quick translation into real-life scenarios that can be used to derive recommendations for cancer survivors. Moreover, we assessed studies conducted in a wide variety of settings, and hence we were able to summarise and report associations between dietary patterns and different cancer prognostic outcomes separately, by specific dietary pattern, outcome and cancer type.

A limitation of this systematic review and meta-analysis was that eligible studies were predominantly observational, including, in some instances, several publications based on the same cohort. In general, most studies derived dietary intake from a single FFQ, although a few used data accumulated from multiple dietary assessments. Additionally, the small number of studies that investigated a common dietary pattern and outcome in a cohort of survivors of the same cancer type limited our ability to conduct meta-analyses to estimate the pooled effect across included studies for tumours other than breast cancer. Similarly, we were unable to perform a meta-analysis across randomised controlled trials, including three pilot studies, owing to heterogeneity between the instruments used for quality of life assessment, which was the most common outcome.

## 5. Conclusions and Final Remarks

An overview of the results reveals that the majority of dietary patterns characterised by a ‘high quality’ diet, often defined according to existing guidelines, as well as *a priori* patterns defined as nutritionally ‘healthy’, can be associated with improved survival in breast and colon cancer survivors. Despite the assumption that dietary patterns are intended to evaluate diet quality as a whole and are a holistic approach to nutrition, this is to some extent, an expected result, which basically leaves us in the same situation already pointed out for nutritional recommendations [8]: we may end up with a tendency to use cancer prevention guidelines for cancer survivors. In this context, a promising approach could be the assessment of dietary patterns directly related to underlying mechanisms linking nutrition factors to cancer progression [10]. Dietary patterns based on biological processes assume that mechanisms underlying the associations between a dietary pattern and cancer are likely due to the individual or synergistic effects of the various dietary components of this pattern. Indeed, accumulating evidence suggests that diet can modulate these mechanisms. Several interrelated biological processes have been proposed, including antioxidant capacity, hyperinsulinemic potential, metabolic or hormonal disruption, and inflammation and immune function [74,75,76,77].

Most randomised trials included in this review evaluated quality of life as primary or secondary outcome related to prognosis. Overall, we may conclude that most dietary interventions tend to improve quality of life and some specific quality of life components among breast cancer survivors. It must be kept in mind, however, that in many instances the effect of diet cannot be assessed independently, as most interventions combined diet and physical activity. However, differences in study design and tools used for quality of life assessment did not allow us to calculate an overall estimate for each specific cancer. Therefore, one of the key issues arising from of this review is the recommendation that future trials evaluating quality of life always include one of the questionnaires widely validated and accepted by most researchers, regardless of the specific aspects and dimensions of quality of life of interest in this particular investigation. On the other hand, there is still need of large, prospective, randomised intervention trials to generate data demonstrating improvements in cancer-specific outcomes (recurrence, disease-free survival) as a result of these dietary (and other lifestyle) interventions. It has long been recognised that such kind of trials are resource- and time-intensive [78]. Since evaluating the impact of lifestyle interventions on survival and cancer-related events requires long follow-up of participants, usually accompanied by a high economic burden, a potential alternative is the assessment of short- and medium-term outcomes of changes in prognostic-related markers. This needs, additionally, further research addressed to assess biomarkers with potential prognostic value (epigenetic, metabolic, and molecular) susceptible to modification by diet and other lifestyle factors.

## Figures and Tables

**Table 1:** Characteristics of the included prospective cohort studies (*n* = 35) examining the association between dietary patterns and prognosis in cancer survivors.

Author, Year	Country	Population, Cohort	Dietary Assessment	Dietary Patterns	Outcomes	Results—Multivariate Adjusted RR/HR(95% CI)	Observations
Several tumour sites							
Inoue-Choi, 2013	USA	IWHS, 2017 cancer cases: breast (*n* = 938), colorectal (*n* = 380), gynaecologic (*n* = 262) and other cancer (*n* = 437), mean age 78.9 years, mean follow-up 5.4 years.	Post-diagnostic 127-items FFQ.	WCRF/AICR guidelines scores.	All-cause mortality, cancer-specific mortality, CVD-specific mortality.	Q4 vs. Q1. All survivors: All-cause mortality: HR = 0.67 (0.49–0.90), p-trend = 0.03; Cancer-specific mortality HR = 0.63 (0.39–1.04), p-trend = 0.21; CVD-specific mortality: HR = 0.92 (0.57–1.47), p-trend = 0.40. Gynaecological cancers: All-cause mortality: HR = 0.96 (0.34–2.69), p-trend = 0.94; Gynaecological cancer-specific mortality: NA; CVD-specific mortality: HR = 1.05 (0.27–4.15), p-trend = 0.83. Other cancer: All-cause mortality: HR = 0.55 (0.30–1.01), p-trend = 0.12.	Gynaecology included cervical, endometrial, ovarian and other female genital organ cancers. ‘Other cancer’ category was not further defined. Models adjusted for age, total number of comorbid conditions (accumulated, 1986–2004), perceived general health and current smoking, cancer stage, cancer type, cancer treatment (surgery, chemotherapy), subsequent cancer diagnosis before 2004, current cancer treatment and person-years since cancer diagnosis. Mean time since cancer diagnosis is 8.6 years (SD = 4.8 years).
Karavasiloglou, 2019	USA	120 gynaecological cancers: ovarian (*n* = 19), cervical (*n* = 54), and uterine cancer (*n* = 47), NHANES III, mean follow-up 12.4 years.	Post-diagnostic 24-h dietary recall.	HEI and MDS.	All-cause mortality.	By 1-unit increase, HEI: HR = 0.92 (0.89–0.96). MDS: HR = 0.77 (0.57–1.04). Good (≥70) vs. Poor (<70) HEI: 0.20 (0.10–0.43). Adherers (5–9) vs. Non-adherers (0–4) MDS: HR = 0.49 (0.18–1.37).	Usual variables of adjustment; alcohol was not included in the adjustment of the MDS model (it is one of the MDS items). Information regarding disease severity or treatment was not available. Important: mean time between diagnosis and completion of the questionnaire is 10.4 years; therefore, these associations refer to long-term survivors.
Breast cancer (BC)							
Kim, 2011	USA	2729 postmenopausal BC stage I-III), NHS study, follow-up 6 years.	Pre- and post-diagnosis FFQ every 4 years (initially 61-items, until 130-items).	Diet quality indices: AHEI, DQIR, RFS, aMED	All-cause mortality, BC-specific mortality and non-BC mortality, BC-recurrence	Q5 vs. Q1 (post-diagnostic diet): All-cause mortality: HEI, RR = 0.85 (0.63–1.17); DQIR, RR = 0.78 (0.58–1.07); RFS, RR = 1.03 (0.74–1.42); aMED, RR = 0.87 (0.64, 1.17). BC-specific mortality: RFS, RR = 1.54 (0.95–2.47) p-trend = 0.02. Distant recurrences: RFS, RR = 1.45 (0.94–2.23) p-trend = 0.001. Pre-diagnostic diet quality indices were not associated with outcomes.	For pre-diagnosis diet, diet quality indices based on a single dietary questionnaire were not associated with total mortality, breast cancer mortality, distant recurrences or non-breast cancer mortality (data not reported). Adjustment for relevant variables.
George, 2011	Mexico, USA	HEAL Study; 670 local or regional BC survivors, follow-up 6 years.	Post-diagnostic 122-items self-administered FFQ 6 and 30-month.	HEI-2005.	All-cause and BC-specific mortality.	Q4 vs. Q1: all-cause mortality HR = 0.40 (0.17–0.94), BC-specific mortality HR = 0.12 (0.02–0.99). All-cause mortality in active-higher HEI-2005 vs. inactive-lowest HEI-2005: HR = 0.11 (0.04–0.36); BC-specific mortality in active-higher vs. inactive-lowest HEI-2005: HR = 0.09 (0.01–0.89).	Adjusted for energy, physical activity, race, stage and tamoxifen use.
Vrieling, 2013	Germany	2522 postmenopausal BC stage I–IV, median follow-up 5.5 years, MARIE study.	1-year pre-diagnostic 176-item FFQ.	Dietary patterns: ‘healthy’ and ‘unhealthy’; defined by principal components and factor analysis.	Overall mortality, BC-specific and non-BC mortality; recurrence of breast cancer.	Q4 vs. Q1 ‘unhealthy’ pattern: HR = 3.69 (1.66–8.17) p-trend < 0.001 (non-BC mortality), HR = 1.34 (0.93–1.94) p-trend = 0.03 (overall mortality), HR = 0.99 (0.64–1.52) p-trend = 0.59 (BC-mortality). Within cases stage I-IIIa, ‘healthy’ pattern HR = 0.74 (0.47–1.15) p-trend = 0.02 (overall mortality), HR = 0.71 (0.48–1.06) p-trend = 0.02 (recurrence).	BMI and physical activity not included in multivariate models.
Inoue-Choi, 2013	USA	IWHS, 938 BC cases.	Post-diagnostic 127-items FFQ.	WCRF/AICR guidelines scores.	all-cause mortality, BC-specific mortality, CVD-specific mortality	Q4 vs. Q1. All-cause mortality: HR = 0.61 (0.39–0.96), p-trend = 0.01. BC-specific mortality: HR = 0.88 (0.41–1.91), p-trend = 0.65. CVD-specific mortality: HR = 0.67 (0.33–1.37), p-trend = 0.10.	Models adjusted for age, total number of comorbid conditions (accumulated, 1986–2004), perceived general health and current smoking, cancer stage, cancer type, cancer treatment (surgery, chemotherapy), subsequent cancer diagnosis before 2004, current cancer treatment and person-years since cancer diagnosis. No data on cancer stage, mean age of cases and mean/median follow-up time. See note in ‘Several tumour sites’ section for this article.
Izano, 2013	USA	NHS, 4103 BC cases stages I-III, median follow-up 9.3 years.	At least 12 months after diagnostic, FFQ	DASH, AHEI-2010.	Primary: BC-mortality; Secondary: distant BC recurrence, non-BC mortality, total mortality.	Q5 vs. Q1 dietary pattern; BC mortality, DASH RR = 0.85 (0.61–1.19) p-trend = 0.47; AHEI-2010 RR =1.07 (0.77–1.49) p-trend = 0.95. Non-BC mortality, DASH RR = 0.72 (0.53–0.99) p-trend = 0.03; AHEI-2010 RR = 0.57 (0.42–0.77) p-trend < 0.0001.	No association with BC recurrence (data not shown) in multivariate models. Results for total mortality (one of the secondary endpoints) not reported, only mentioned in methods. Adjustment: age at diagnosis, energy intake, BMI, smoking and physical activity.
George, 2014	USA	2317 postmenopausal women invasive BC (localised, regional, distant, unknown), (50–79 years), WHI Dietary Modification Trial (*n* = 1205) and Observational Study (*n* = 1112), follow-up 9.6 years.	Post-diagnostic, self-administered FFQ at baseline and at 3-year of follow-up.	HEI-2005 scores.	All-cause and cause-specific mortality.	Q4 vs. Q1 HEI score; all-cause mortality HR = 0.74 (0.55–0.99) p-trend = 0.04; non-BC mortality HR = 0.58 (0.38–0.87) p-trend = 0.01; BC mortality HR = 0.91 (0.60–1.40) p-trend = 0.63.	Multivariate model not adjusted for BMI and smoking status. Further adjustment for BMI did not modify HRs (data not reported).
McCullough, 2016	USA	4452 cases (40–93 years), CPS-II Nutrition Cohort, mean follow-up 9.8 years.	Pre- and post-diagnostic 68-item Block FFQ (baseline), 152-item Harvard FFQ twice during follow-up.	Dietary pattern scores based on ACS dietary guidelines.	All-cause mortality and deaths from BC, non-BC and CVD.	Highest vs. Lowest post-diagnostic dietary pattern: BC-mortality RR = 1.44 (0.90–2.30); CVD mortality RR = 0.81 (0.47–1.39); Non-BC mortality RR = 0.78 (0.56–1.07) p-trend = 0.03 & per 2-point increase RR = 0.88 (0.79–0.99). Pre-diagnostic diet score was not associated with all-cause mortality.	Adjustment for usual variables; alcohol not included in the final model since it did not change the estimated RRs.
Jang, 2018	Korea	511 cases (mean age 51.9 years), mean follow-up 69 months, Hanyang University Seoul Hospital.	Post-diagnostic 24-h diet recall.	DII (34 items).	BC recurrence and overall mortality.	Q4 vs. Q1; BC recurrence HR = 2.3 (1.17–4.71) p-trend = 0.019; overall mortality HR = 3.0 (1.08–8.83) p-trend = 0.041.	Not adjusted for physical activity, alcohol and smoking status. Associations were also significant among women < 50 y, premenopausal, BMI ≥ 25 kg/m^2^, HR+ tumours, tumour size > 2 cm and lymph node metastasis (strata of prognostic factors).
Sun, 2018	USA	2295 postmenopausal women (50–79 years at recruitment), invasive BC, 12 years follow-up, WHI study.	Pre- and post-diagnostic FFQ, HEI-2010 based on 12 components.	HEI-2010 score.	All-cause mortality, BC-mortality, non-BC mortality.	Compared with women with stable diet quality, women who decreased ≥15% HEI-2010, HR = 1.66 (1.09–2.52) for BC-mortality. Women who increased ≥15% HEI-2010 vs. stable diet quality HR = 1.00 (0.81–1.23) for all-cause mortality, HR = 0.98 (0.67–1.44) for BC-mortality and HR = 0.96 (0.74–1.23) for other causes.	Adjustment for relevant variables.
Zheng, 2018	USA	2150 postmenopausal women (age 50–79 years), 13.3 years follow-up, WHI.	1.5 years post-diagnostic: FFQ 120-items plus other related questions.	E-DII (32 components).	All-cause, BC-specific, and CVD mortality.	Q1 vs. Q4 E-DII; HR = 0.96 (0.62–1.49) p-trend = 0.96 (BC mortality); HR = 0.82 (0.63–1.05) p-trend = 0.17 (all-cause mortality); HR = 0.44 (0.24–0.82) p-trend = 0.005 (CVD mortality).	Adjustment for usual variables except for alcohol (probably because alcohol is one of the DII’s items). Stratified analyses for hormonal receptors (ER, PR and combined ER-PR status) with no significant interactions.
Karavasiloglou, 2019	USA	110 women, NHANES III, mean follow-up 8.6 years.	Post-diagnostic 24-h dietary recall.	HEI, MDS.	All-cause mortality.	By 1-unit increase, HEI: HR = 0.97 (0.95–0.99); MDS: HR = 0.97 (0.82–1.16). Good (≥70) vs. Poor (<70) HEI: 0.49 (0.25–0.97). Adherers (5–9) vs. Non-adherers (0–4) MDS: HR = 0.78 (0.47–1.32).	Usual variables of adjustment; alcohol was not included in the adjustment of the MDS model (it is one of the MDS items). Information regarding disease severity or treatment was not available. See note in ‘Several tumour sites’ section for this article.
Wang, 2020	China	3450 cases stage I-IV, SBCSS, follow-up time 8 years.	Post-diagnostic: 93-item semi-quantitative FFQ at 5 years.	CHFP-2007, CHFP-2016, modified DASH, HEI-2015.	All-cause mortality, BC-specific mortality, BC-specific events.	Q1 vs. Q4 dietary pattern; all-cause mortality: CHFP-2007 HR = 0.66 (0.48–0.89), CHFP-2016 HR = 0.75 (0.55–1.01), DASH HR = 0.66 (0.49–0.91). BC-specific events: CHFP-2007 HR = 0.64 (0.44–0.93), CHFP-2016 HR = 0.67 (0.45–0.99), DASH HR = 0.60 (0.40–0.90). Similar association patterns observed for BC-specific mortality.	BC-specific events defined as recurrence or metastasis of BC and deaths from BC. Usual variables of adjustment except for alcohol (not included). Information on outcomes collected during the 10-year post-diagnosis by means on in-person survey.
Wang, 2021	USA	8482 BC cases stage I-III, median follow-up 14 years, NHS and NHSII.	Post-diagnostic semi-quantitative FFQ every 4 years.	DRRD (9 components).	All-cause mortality, BC-specific mortality.	Q5 vs. Q1 DRRD; BC-mortality: HR = 0.80 (0.65–0.97) p-trend = 0.02; all-cause mortality HR = 0.66 (0.58–0.76) p-trend < 0.0001. Compared with lower score (≤median) before & after diagnosis, women whose score improved from low to high: HR = 0.77 (0.62–0.95) for BC-specific mortality; HR = 0.85 (0.74–0.97) for overall mortality.	Multivariate model adjusted for key confounders. Included change in BMI from pre- to post-diagnostic in adjustments.
Colorectal cancer (CRC)							
Inoue-Choi, 2013	USA	IWHS, 380 CRC cases, older female survivors (no age specified).	Post-diagnostic 127-items FFQ.	WCRF/AICR guidelines scores.	All-cause mortality, CRC-specific mortality.	Q4 vs. Q1. All-cause mortality: HR = 1.19 (0.59–2.43), p-trend = 0.64. CRC-specific mortality: HR = 1.16 (0.33–4.12), p-trend = 0.84. CVD-specific mortality: HR = 2.61 (0.78–8.71), p-trend = 0.19.	Models adjusted for age, total number of comorbid conditions (accumulated, 1986–2004), perceived general health and current smoking, cancer stage, cancer type, cancer treatment (surgery, chemotherapy), subsequent cancer diagnosis before 2004, current cancer treatment and person-years since cancer diagnosis. No data on cancer stage, mean age of cases and mean/median follow-up time. See note in ‘Several tumour sites’ section for this article.
Zhu, 2013	Canada	529 invasive CRC, Newfoundland Familial Colorectal Cancer Registry, median follow-up 6.4 years.	Pre-diagnostic semi-quantitative 170-items FFQ (including vitamin and dietary supplements). Principal factor analysis (39 food groups).	Dietary patterns extracted: ‘processed meat pattern’, ‘prudent vegetable pattern’ and ‘high-sugar pattern’.	Disease-free survival (DFS) and overall survival (OS).	Q4 vs. Q1: processed meat pattern CRC HR = 1.82 (1.07–3.09), p-trend = 0.09 for DFS. Colon HR = 2.29 (1.19–4.40) & rectum HR = 0.97 (0.38–2.45) for DFS. Colon HR = 2.13 (1.03–4.43) for OS.	Physical activity, alcohol and smoking status not included in the adjustment.
Pelser, 2014	USA	NIH-AARP Diet and Health study, 4213 colon and 1514 rectal cancer cases, 5 years follow-up.	Pre-diagnostic 124-item FFQ.	HEI-2005.	All-cause, CRC-mortality and CVD-mortality.	Q5 vs. Q1; Colon cancer: all-cause mortality: RR = 0.95 (0.78–1.16), p-trend = 0.22; CRC-mortality RR = 0.99 (0.77–1.27), p-trend = 0.41; CVD-mortality RR = 0.45 (0.23–0.87), p-trend = 0.01. Rectal cancer: all-cause mortality: RR = 0.60 (0.42–0.86), p-trend = 0.04; CRC-mortality RR = 0.64 (0.41–0.99), p-trend = 0.05; CVD-mortality RR = 0.28 (0.06–1.43).	Variables of adjustment usually used except for socioeconomic status.
Fung, 2014	USA	1201 stage I–III CRC cases (women only), median follow-up 11.2 years, NHS.	Post-diagnostic: FFQ at least 6 months after diagnostic; principal component analysis.	AHEI-2010, aMED and DASH and 2 derived dietary patterns: western and prudent diet.	Overall and CRC-specific mortality.	Q5 vs. Q1; AHEI-2010: Overall mortality: HR = 0.71 (0.52–0.98), p-trend = 0.01; CRC mortality: HR = 0.72 (0.43–1.21), p-trend = 0.07.	No other diet quality score or dietary pattern was associated with overall or CRC-specific mortality.
Romaguera, 2015	Europe (10 countries)	EPIC, 3292 CRC cases, mean follow-up 4.2 years.	Pre-diagnostic country-specific validated dietary questionnaires and standardised EPIC Nutrient Data Base.	WCRF/AICR guidelines. Score range 0–6 in men, 0–7 in women; higher scores: greater adherence.	CRC-specific and overall mortality.	CRC-specific mortality: 2nd, 3rd and 4th concordance with recommendations vs. lowest concordance: HR_2nd_ = 0.87 (0.72–1.06), HR_3rd_ = 0.74 (0.61–0.90), HR_4th_ = 0.70 (0.56–0.89); p-trend < 0.0001. Similar results for overall survival (p-trend 0.004).	Adjusted by usual variables including smoking. Body fatness, PA and alcohol were part of the WCRF score, so these were not included in the adjustment.
Jacobs, 2016	USA	MEC study, 4204 cases (men and women aged 45–75 years), stage: localised, regional, distant or unknown, mean follow-up 6.0 years.	Pre-diagnostic FFQ (>180 food items).	4 diet quality indexes: HEI-2010, AHEI-2010, aMED and DASH.	CRC-specific and all-cause mortality.	African-American women: aMED, CRC-specific mortality: HR_1SD_ = 0.86 (0.77–0.96); aMED, all-cause mortality: HR_1SD_ = 0.88 (0.81–0.96). No significant for men in either case. HEI-2010, AHEI-2010, and DASH no significantly associated with CRC-specific or all-cause mortality.	Usual variables of adjustment used except for alcohol since it is part of some scores.
Yuan, 2017	USA	2006 cases from 2 cohorts: NHS, and HPFS, 12.7 years median follow-up	Post-diagnostic FFQ every 4 years	Two dietary insulin (DI) scores: DI-index and DI-load.	CRC-specific mortality and overall mortality.	Q5 vs. Q1. CRC-specific mortality: DI-load HR = 1.82 (1.20–2.75), p-trend = 0.006 & DI-index HR = 1.66 (1.10–2.50), p-trend = 0.004. Overall mortality: HR = 1.33 (1.03–1.72), p-trend = 0.03 for DI-load & HR = 1.32 (1.02–1.71), p-trend = 0.02 for DI-index. In BMI ≥ 25 HR = 2.32 (1.21–4.46) for higher DI-index; BMI ≥ 25 kg vs. BMI < 25 (p-interaction = 0.01).	Usual variables of adjustment used (BMI, PA, alcohol, smoking status).
Ratjen, 2017	Northern Germany	1404 CRC cases, median follow-up 7 years, median age 69 years, 56% men, PopGen biobank.	Post-diagnostic, 112-item semi-quantitative FFQ.	Two a priori-defined dietary patterns: MMDS and HNFI.	All-cause mortality.	MMDS: HR_Q4-Q1_ = 0.48 (0.32–0.74) & HR_1-point increment_ = 0.88 (0.81–0.96), p-trend = 0.003. HNFI: HR_Q4-Q1_ = 0.63 (0.39–1.04) and HR_1-point increment_ = 0.90 (0.82–0.99), p-trend = 0.04.	Usual variables of adjustment used. No information available for CRC-specific mortality.
Sharma, 2018	Canada	532 CRC (mean age 60 years), mean follow-up 6.27 years, Newfoundland Familial Colorectal Cancer Registry (NFCCR).	Pre-diagnostic 169-item FFQ.	Cluster Analysis (CA), Principal Component Analysis (PCA), altMED, RFS and DII scores.	Overall mortality (OM) and combined Mortality, Recurrence or Metastasis (cMRM).	For cMRM: PCA-processed meats HR = 1.82 (1.07–3.09); CA-meat & dairy products HR = 2.19 (1.03–4.67); CA-total grains, sugar, soft drinks HR = 1.95 (1.13–3.37). For OM: Poor adherence aMED HR = 1.62 (1.04–2.56). No association with OM/cMRM with prudent vegetable, high sugar pattern, RFS and DII.	Usual variables of adjustment used.
Zheng, 2020	USA	WHI, 463 CRC cases postmenopausal women (aged 50–79 years), 11.6 years follow-up	Post-diagnostic FFQ (number of items not reported).	E-DII (31 components); DII calculated from diet plus supplements and from diet only.	All-cause, total cancer, and CRC-specific mortality.	T1 vs.T3: E-DII (diet + supplements) HR = 0.49 (0.31–0.79) for all-cause mortality; HR = 0.57 (0.29–1.10) for total cancer mortality; HR = 0.58 (0.28–1.22) for CRC-specific mortality. E-DII (diet only) HR = 0.72 (0.46–1.12) for all-cause mortality.	Most pro-inflammatory E-DII (T3) as ref. E-DII score from diet plus supplements and from diet only were both examined. Models not adjusted for alcohol consumption probably because alcohol is one of the items of DII.
Tabung, 2020	USA	1718 stage I–III CRC, NHS and HPFS cohorts, follow-up 9.9 years.	Pre- and post-diagnostic FFQ (number of items not reported).	EDIH score.	CRC-specific mortality and all-cause mortality.	Q5 vs. Q1; Pre-diagnostic EDIH: HR = 1.66 (1.03–2.69) for CRC-mortality & HR = 1.24 (0.97–1.58) for all-cause mortality. Higher EDIH pre- & post-diagnostic HR = 1.51 (0.98–2.32) for CRC-mortality & HR = 1.31 (1.04, 1.64) for all-cause mortality.	Usual variables of adjustment used.
Prostate cancer (PC)							
Kenfied, 2014	USA	4538 non-metastatic PC, HPFS, median follow-up (8.9 years for lethal and 9.1 years for fatal outcomes).	Post-diagnostic 130-items FFQ.	Med-Diet adherence.	PC-specific and overall mortality.	High vs. low adherence: HR = 0.98 (0.75–1.29) for lethal disease; HE = 1.01 (0.75–1.38) for fatal disease; HR = 0.78 (0.67–0.90), p-trend = 0.0007 for overall survival.	Assessed traditional and alternative Mediterranean diet pattern. Usual variables of adjustment used.
Yang M, 2015	USA	926 cases non-metastatic PC, PHS I or II, follow-up median 13.8 years.	Post-diagnostic FFQ (number of items not reported).	Prudent and Western pattern.	PC-specific and overall mortality.	Q4 vs. Q1: Western HR = 2.53 (1.00–6.42), p-trend = 0.02 for PC-mortality & HR = 1.67 (1.16–2.42), p-trend = 0.01 for all-cause mortality. Prudent HR = 0.64 (0.44–0.93) p-trend = 0.02 for all cause-mortality.	Usual variables of adjustment used.
Zucchetto, 2016	Italy	726 cases (median age 66 years), median follow-up 12.7 years, cohort study from a case-control study.	Pre-diagnostic 78-items + common Italian recipes FFQ.	DII (31 items).	All-cause and PC-specific survival.	T3 vs.T1: DII HR = 1.25 (0.86–1.83) for all-cause mortality. Heterogeneity to Gleason score p < 0.01. Gleason score 7–10 Pca, DII HR= 2.78 (1.41–5.48) for all-cause & HR = 4.01 (1.25–12.86) for PC-specific mortality.	Model adjusted for area of residence, calendar period of diagnosis, age at diagnosis, education, smoking habits, abdominal obesity, alcohol intake and energy intake.
Head and Neck cancer							
Arthur, 2013	USA	542 cases head and neck squamous cell carcinoma (HNSCC); mean age 59 years, mean follow-up ~6 years.	Pre-treatment self-administered, semi quantitative Harvard FFQ (131-item); principal component analysis.	Two dietary patterns: whole-foods pattern, western pattern.	Recurrence and all-cause survival.	Most adherence to the whole-foods pattern HR_Q5vsQ1_= 0.56 (0.34–0.92), p-trend = 0.01.	Limitation: the heterogeneous nature of the study population regarding tumour site. Multivariate models adjusted for age, sex, tumour site, cancer stage, treatment, ACE-27 comorbidities, smoking, BMI and total energy intake.
Crowder, 2019	USA	336 cases, University of Michigan Head and Neck Specialised Program of Research Excellence, follow-up 1 year.	Pre-treatment self-administered 2007 Harvard FFQ.	Principal component analysis, 2 dietary patterns: prudent and western.	Nutrition impact symptoms (NIS) 1-year post-diagnostic: difficulty chewing, dysphagia-liquids, dysphagia-solids foods, mucositis.	Prudent pattern: difficulty chewing OR = 0.44 (0.21–0.93), p-trend = 0.03; dysphagia liquids OR = 0.38 (0.18–0.79), p-trend = 0.009; dysphagia solid foods OR = 0.46 (0.22–0.96), p-trend = 0.03; mucositis OR = 0.48 (0.24–0.96), p-trend = 0.03, NIS summary score OR = 0.45 (0.22–0.94), p-trend = 0.03.	NIS were measured using the UM Head and Neck Quality of Life questionnaire. Final multivariable models not adjusted for PA or alcohol.
Ovarian cancer (OC)							
Thomson, 2014	USA	636 cases (postmenopausal, mean age 63 years), WHI, follow-up time not found or not clear.	Pre-diagnostic FFQ (number of items unknown).	HEI-2005 score.	Overall and OC-specific mortality.	For all-cause mortality: HEI-2005 HR_T3-T1_ = 0.73 (0.55–0.97), p-trend = 0.03. For OC-mortality: HEI-2005 HR_T3-T1_ = 0.75 (0.55–1.01), p-trend = 0.06. Women with waist ≤88 cm and no history of diabetes: HR = 0.73 (0.54–0.98).	No adjustments for smoking status, alcohol and BMI.
Hansen, 2020	Australia	OPAL study, 958 cases before diagnosis (n = 678) median follow-up 3.9 years and post-diagnosis (n = 512), median follow-up 3.5 years.	Collected at baseline, 12 and 24 months using a validated semi quantitative FFQ.	Pre- and post-diagnostic Healthy lifestyle index (HLI): including smoking status, physical activity, BMI, alcohol, diet quality score.	Overall survival.	HLI pre-diagnostic: HR most vs. least healthy HR = 0.79 (0.59–1.04). HLI Post-diagnosis most vs. least healthy HR = 0.61 (0.40–0.93). Diet quality score Pre-diagnostic HR_T3-T1_ = 0.99 (0.76–1.31) p-trend = 0.9. Post-diagnostic diet quality score HR_T3-T1_ (best quality vs. worst) = 1.01 (0.63–1.60), p-trend = 0.9.	Pre-diagnostic models: adjusted for age, education and comorbidities. Post-diagnostic models: adjusted for age, education, comorbidities, stage of disease at diagnosis, histological subgroup and residual disease remaining after surgery. Diet quality score based on WCRF/AICR guidelines (excluding alcohol).
Bladder cancer							
Westhoff, 2018	USA	595 non-muscle-invasive cancer (non-Hispanic white), University of Texas M.D. Anderson Cancer Center, Scott Department of Urology, median follow-up 65.7 months.	Pre-diagnostic semi-quantitative 181-items FFQ, exploratory factor analysis (included 135 items).	4 dietary patterns derived: fruits and vegetables, western, low-fat, and Tex-Mex.	Recurrence or progression to muscle-invasive bladder cancer or metastatic tumours.	T3 vs. T1; Recurrence, Western HR = 1.48 (1.06–2.06), p-trend = 0.03. Progression, Western HR = 1.56 (0.91–2.65) p-trend = 0.10. No significant associations with risk of recurrence or progression found for the other patterns.	Models adjusted for age, sex, education, income, BMI, smoking status and intensity, total energy intake, grade, tumour multiplicity, concomitant carcinoma in situ and treatment.
Multiple myeloma							
Lee, 2020	USA	423 cases (mean age 70–72 years women-men), NHS and HPFS, follow-up median 3.5 years.	Pre-diagnostic 130-items FFQ.	AHEI-2010, aMED, DASH, Prudent, Western and EDIR/EDIP/EDIH.	Multiple myeloma-specific mortality, all-cause mortality.	1-SD increase; Specific mortality: AHEI-2010 HR = 0.76 (0.67–0.87), *p* < 0.001; aMED HR = 0.85 (0.75–0.97), *p* = 0.01; DASH HR = 0.85 (0.76–0.95), *p* = 0.006; Prudent pattern HR = 0.76 (0.66–0.87), *p* < 0.001; Western pattern HR = 1.24 (1.07–1.44), *p* = 0.005; EDIR HR = 1.16 (1.02–1.33), *p* = 0.03; EDIH HR = 1.17 (1.01–1.35), *p* = 0.03. Similar results for all-cause mortality.	No adjustments for smoking status, alcohol and physical activity.

Abbreviations: CI, confidence interval; WCRF/AICR, World Cancer Research Fund/American Institute for Cancer Research; ACS, American Cancer Society; RR, relative risk; HR, hazard ratio; ER, oestrogen receptor; PR, progesterone receptor; CVD, cardiovascular. Study names: WHI, Women’s Health Initiative; UM HN-SPORE, University of Michigan Head and Neck Specialised Program of Research Excellence; CPS-II, Cancer Prevention Study II; CALGB, National Cancer Institute–sponsored Cancer and Leukaemia Group B; SBCSS, Shanghai Breast Cancer Survival Study; LACE, Life After Cancer Epidemiology; NHS, Nurses’ Health Study; MEC, Multiethnic Cohort; IWHS, Iowa Women’s Health Study; WHS, Women’s Health Study; HPFS, Health Professionals Follow-up Study; NCI, National Cancer Institute; CWLS, Collaborative Women’s Longevity Study; HEAL, Health, Eating, Activity, and Lifestyle; LIBCSP, Long Island Breast Cancer Study Project; CBCS, Carolina Breast Cancer Study; AOCS, Australian Ovarian Cancer Study; CaPSURE, Cancer of the Prostate Strategic Urologic Research Endeavor; RFS, Recommended Food Score; OPAL, Ovarian cancer Prognosis And Lifestyle; NSHD, Northern Sweden Health and Disease Study; DDCH, Danish Diet, Cancer and Health Study; NOWAC, Norwegian Women and Cancer; PHS, Physicians’ Health Study; DACHS, Darmkrebs: Chancen der Verhütung durch Screening; BCPP, Bladder Cancer Prognosis Programme. Dietary patterns: HEI, Healthy Eating Index; DASH, Dietary Approaches to Stop Hypertension; AHEI, Alternative Healthy Eating Index; DII, Dietary Inflammatory Index; E-DII, Energy-Adjusted Dietary Inflammatory index; DQIR, Diet Quality Index-Revised; RFS, Recommended Food Score; EDIR, Empirical Dietary Index for Insulin Resistance; EDIP, Empirical Dietary Inflammatory Pattern; EDIH, Empirical Dietary Index for Hyperinsulinemia; MMDS, Modified Mediterranean Diet Score; HNFI, Healthy Nordic Food Index; CHFP, Chinese Food Pagoda.

**Table 2:** Characteristics of the included randomised controlled trials (*n* = 14) examining the association between dietary interventions and prognosis in cancer survivors.

Author, Year	Country	Population (Clinical Features, Sample Size, Age, Follow-Up)	Intervention Description	Outcome (Primary, Secondary)	QoL Assessment	Results: Effect Parameter (CI or *p*-Value)	Observations
Several cancers							
Yun, 2017	South Korea	Cancer survivors who had completed primary cancer treatment within the last 18–24 months. 248 participants randomised: 88 allocated to usual care, 166 to intervention.	LEACH program: first 1-h health education workshop (physical activity, dietary habits, and distress management) and a 3-h leadership workshop. Next individual coaching by telephone for a 24-week period; overall 16 sessions of tele-coaching were conducted: 30 min per week for 12 sessions, 30 min per 2 weeks for 2 sessions and 30 min per month for 2 sessions. Total duration: 1 year.	Primary: changes in physical activity, diet and in PTGI. Secondary: quality of life (QoL).	HADS, EORTC QLQ-C30.	Assessment at 12-month, adjusted means intervention group vs. control group (*p*-value): PTGI: 66.3 vs. 61.2 (*p* = 0.065). HADS: 5.2 vs. 5.7 (*p* = 0.23). EORTC (global health): 69.0 vs. 66.0 (*p* = 0.27). EORTC (fatigue): 34.8 vs. 41.9 (*p* = 0.01).	Included in situ, localised or regional with a favourable prognosis of cancers of the breast, stomach, colon and lung. The assessment at 12-months was carried out over 72 subjects (control group) and 134 (intervention group).
Breast cancer (BC)							
Scott, 2013	UK	90 women with early stage cancer (stage I–III), treated within the previous 3–18 months; mean age 56 years. 47 intervention, 43 controls; completed assessment at 6-month: 41 and 48.	6-month lifestyle intervention: exercise + hypocaloric healthy eating program: 3 supervised exercise sessions/week and individualised dietary advice + weekly nutrition seminars. Diet sessions: information on portion sizes from common foods and healthy eating plan. Goal: to reduce 600 kcal of daily calorie intake of their calculated energy requirements.	Primary: body weight, body composition. Secondary: quality of life (QoL).	FACT-B assessed at baseline and at 6-month.	FACT-B QoL: significant improvement in the intervention group: >6 points (*p* = 0.004) in FACT-B score and >2 points (*p* = 0.007) in the breast cancer subscale. Moreover, reduction in the intervention group of waist circumference (*p* < 0.001) and waist-to-hip ratio (*p* < 0.005).	
Goodwin, 2014	USA and Canada	LISA Study. Multicentre randomised trial in postmenopausal women with tumours stage T1-3N0-3M0, BMI ≥ 24. Lifestyle intervention (up to 24 mo) diet + physical activity counselling, evaluating secondary outcomes. Groups: (*n* = 167) mail-based intervention and (*n* = 171) individual lifestyle intervention (LI).	Both arms received information on healthy lifestyle at baseline and at 1-year. Individualised LI: 2-year telephone-based intervention on the diabetes prevention program. Goal: 10% weight loss to a BMI not less than 21; calorie reduction to attain 500–1000 kcal daily deficit, and reduction in fat to 20% of kcal, and increased intake of fruits, vegetables, and grains; gradual increase in moderate-intensity aerobic physical activity to 150–200 min/week.	Primary: disease-free survival. Secondary: overall survival, distant-disease-free survival, weight loss, quality of life (QoL).	QoL: EORTC QLQ-C30 (physical condition and overall QoL score); SF-36 (PCS and MCS); Fatigue Symptom Inventory; Breast Symptom Checklist.	Weight: mean weight loss was significantly (*p* < 0.001) greater in the LI arm vs. comparison arm: 5.3% vs. 0.7% at 6 months, 3.6% vs. 0.4% at 24 months. QoL: mean change in SFS6-PCS from baseline, LI arm vs. comparison arm: 4.2 vs. 2.3 at 6 months, 4.4 vs. 2.9 at 12 months, 4.1 vs. 4.4 at 24 months; *p* = 0.005. No significant changes in SF36-MCS. EORTC QLQ-C30 physical condition score (*p* < 0.001). No significant improvement in EORTC QLQ-C30 Quality of Life Score (*p* = 0.062). All *p*-values are adjusted for time period of assessment.	Accrual was terminated at 338 of 2150 planned patients because of loss of funding. Therefore, only intermediate (24-month) secondary outcomes are presented.
Swisher, 2015	USA	Survivors triple-negative BC (stage I–III), BMI > 25, age < 80 years, average time at enrolment in the study after diagnosis 4–5 years. 28 women enrolled: 20 allocated to control group, 18 to the intervention.	Moderate-intensity aerobic exercise (150 min per week, for 12 weeks) and diet counselling, compared to usual care. Dietary counselling based on 2 individual sessions with the study dietitian; goal: to decrease dietary fat intake by 200 kcal per week.	Primary outcome: weight loss. Secondary: physical function, quality of life (QoL).	FACT-B.	Weight: subject in the intervention lost more body fat (2.4% loss vs. 0.4% gain, *p* < 0.05) than the control group. QoL (FACT-B): improvements in physical well-being (*p* < 0.05) and BC-specific items (*p* < 0.05).	Assessment based upon women who completed the trial (12 weeks): 18 in the intervention group and 10 from the control group.
Demark-Wahnefried, 2015	USA	The ENERGY trial: single-blinded randomised phase 3 trial. Participants: women diagnosed within the previous 5 years on cancer stage-I-III, aged > 21 years and BMI 25–45. Intensive intervention (*n* = 344) or less intensive intervention (control arm) (*n* = 348).	Intervention: group-based, semi-structured weight loss program + telephone counselling and tailored newsletters, according to ACS guidelines. 4 months, 1 h group session/week + 1 session/week for 2 months and 1 session/week during 6–12 months + personalised guidance in between the sessions. + mailed newsletter on a quarterly basis from 6–24 months (individually tailored). Control group received two contacts: at baseline and at 6 months.	Primary outcome: quality of life (QoL).	SF-36; refined Impact of Cancer Scale (IOCv2); BCPT Symptom Scales; CES-D.	Assessment at 12 and 24-month. Non-significant changes for SF36 vitality subscale score (*p*-values 0.509 and 0.185). Improvement (*p* = 0.051) of SF-36 physical function at 12 months and no significant change at 24 months (*p* = 0.185); Greater positive impact of cancer (*p* = 0.046) at 12 months. Depressive symptoms (CES-D) increased at 24 moths (*p* = 0.03).	The SF36 only included specific scales for vitality and physical functioning; the IOCv2 measures impact of cancer on QoL; the BCPT Symptom Scales measures side effects of medical interventions; the CES-D measures depressive symptoms. Unexpected findings related to depressive symptoms.
Kwiatkowski, 2017	France	PACThe trial. Patients enrolled within 9 months after chemotherapy or radiotherapy completion. 251 participants randomised: 117 intervention, 115 control group.	2-week intervention in hydrothermal centres including APANE (adapted physical activity and nutritional education). Energy intake: 1200 kcal/day. Diet program based on Four-Group Point Method. Control group: individual standard recommendations at home.	Primary outcome: long-term (6-month to 5-years) quality of life.	SF36 (global score).	Effect-sizes (difference between means of the two groups divided by the common standard deviation) for the SF36 score at different time periods: 6 months 0.63 (0.37, 0.89); 1 year 0.29 (0.03, 0.55); 2 years 0.27 (−0.01, 0.56). Effect-size over the whole follow-up period 0.33 (0.23, 0.43), *p* < 0.01.	Secondary endpoints: anxiety/depression (HAD), sleep (adapted from Leeds sleep evaluation questionnaire), physical/sedentary activity scores.
Zick, 2017	USA	Pilot study, 30 breast cancer patients stage 0-IIIa (15 intervention, 15 control group)	FRD: rich in fruits, vegetables, whole grains, and omega-3 fatty acid-rich foods. 3-months, phone counselling. Control: 8 sessions general health topics excluding diet).	Primary outcome: fatigue. Secondary: sleep quality.	BFI, PSQI	Adjusted means (difference between baseline and 3-months). BFI decreased by 2.4 in the FRD group vs. controls (*p* = 0.01). PSQI score decreased by 2.5 t in FRD group and increased by 0.9 in the control group (*p* = 0.03).	Intention-to-treat (ITT) analysis. Dietary assessment: at baseline and 3 months by means of day food records and 24-h recalls.
Chlebowski, 2020	USA	WHI-DM trial. 3374 breast cancer survivors (1299 intervention, 2075 controls) median follow-up 19.6-year.	Low-fat dietary pattern: the goals were to reduce fat intake to 20% of energy and increase vegetable, fruit, and grain intake. Intervention period: 8.5-years.	Overall mortality, breast cancer specific mortality.	-	Mortality: HR 0.85 (0.74–0.96), *p* = 0.01. Breast cancer mortality: HR 0.79 (0.64–0.97), *p* = 0.02.	Intention-to-treat, secondary analysis (the primary outcome was recurrence). Lack of breast cancer therapy information.
Ruiz-Vozmediano, 2020	Spain	72 women stage IIA-IIB with treatment completed within previous 12 months. Randomised to intervention (*n* = 36) and control group (*n* = 36), completion of treatment 12 mo earlier. Follow-up: 6 month after intervention.	Intervention (6-month); diet: three 5-h workshops on healthy eating patterns and information on risk factors and prevention; exercise: 7-week period, 60-min class, 3/week, and mindfulness program (4-week, 2/week, 90 min. Control group: usual care.	Primary outcome: quality of life (QoL). Secondary outcome: change in weight.	EORTC QLQ-C30, 5 functional domains: physical, role, cognitive, emotional, and social.	Comparison of means (intervention vs. control at 6-month: significant improvements in physical functioning (*p* = 0.027), role functioning (*p* = 0.028), dyspnoea symptoms (*p* = 0.066). No significant changes in global health and fatigue.	only 15 patients completed at least 75% of program sessions.
Colorectal cancer (CRC)							
Bourke, 2011	UK	Pilot trial; 18 colon cancer survivors, mean age 69 years, Dukes stage A-C, recruited months post-surgery; 9 intervention, 9 controls.	Intervention: 12-week program of home-based exercise sessions and dietary advice (*n* = 9); controls: standard care.	Exercise and dietary behaviours, fatigue and quality of life (QoL).	FACT-F (fatigue) and FACT-C (CRC-specific QoL).	Intervention vs. control: improved fatigue (FACT-F score) *p* = 0.005 and no change in QoL (FACT-C score) *p* = 0.80.	
Bonelli, 2013	Italy	Double-blind, phase III, randomised, placebo-controlled trial. 411 post-polypectomy (within 6 months from enrolment). 200 intervention, 211 placebo group. Median follow-up 4 years.	Active compound (200 μg selenium, 30 mg zinc, 2 mg vitamin A, 180 mg vitamin C, 30 mg vitamin E) vs. placebo; daily, 5 years.	Primary: recurrent adenomas or incident colorectal cancer. Secondary: advanced adenoma.	-	Recurrent adenomas (intervention vs. placebo): HR = 0.61 (0.41–0.92); for small tubular adenomas HR = 0.61 (0.37–0.99); advanced adenomas HR = 0.50 (0.24–1.01).	Intention-to-treat analysis in 330 (out of 411) participants with follow-up colonoscopy (164 intervention and 166 placebo group).
Ho, 2020	China	223 colorectal cancer survivors (82 women), mean age 65 years. 4 groups: Group A (Diet + PA), Group B (Diet only), Group C (PA only), Group D (control group).	Intervention: ‘Moving Bright, Eating Smart’. Reduce red/processed meat to <5 servings/week (<2 servings of processed meat) and to limit refined grains to 2 servings/day. Overall 12-month, with decreasing frequency on contacts along the year. Control: usual care.	Quality of life (QoL); assessment at 6, 12, 18, and 24 months.	SF-12 (health-related QoL), SF-6D utility index, FACT-C (CRC-health related QoL), FACT-G (excluding disease-specific items), HADS (anxiety and depression).	Mean difference between groups, dietary intervention vs. not receiving diet intervention: At 12-mont, SF-6D utility index scores 0.042 (0.003–0.081) and FACT-G total score 3.09 (0.13–6.04). At 24-month, SF-12 PCS scores (2.57 (0.69–4.45) and the FACT-G total scores 3.14 (0.23–6.04). Overall, reduction in HADS-depression 0.71 (1.28–0.14).	Intention-to-treat principle. Results on physical activity intervention available, but no results on combined intervention.
Prostate cancer							
Parsons, 2020	US	Men’s Eating and Living (MEAL) study, 478 men, 50–80 years, with biopsy-proven prostate adenocarcinoma early-stage (cT2a or less and PSA < 10 ng/mL). Intervention (*n* = 237), controls (*n* = 241).	MEAL intervention: counselling behavioural intervention by telephone promoting consumption of 7 or more vegetable servings daily; duration 24 months. Control group: written information about diet and prostate cancer.	Primary: time to progression (by biopsy and PSA changes). Secondary: health related quality of life (QoL).	Several functional and health prostate cancer- related QoL scores.	No significant difference in time to progression (intervention vs. control: adjusted HR 0.97 (0.76–1.25), *p* = 0.84.	Results on QoL no reported.
Endometrial cancer							
Koutoukidis, 2019	UK	DEUS pilot trial: parallel, randomised, controlled pilot trial; 54 survivors stage I-IVA endometrial cancer; allocation to either intervention (*n* = 26) or usual care (*n* = 28).	Intervention: the ‘Shape-Up following cancer treatment’; 8 weeks, group-based weekly 1.5 h sessions on healthy eating and physical activity based on Social Cognitive Theory and Control Theory. Control group: usual care.	Diet, physical activity, body composition, and health-related quality of life (QoL)	EORTC Core 30 and Endometrial Cancer Module (QLQ-EN24)	Change (mean) from baseline to 8 weeks: EORTC QLQ-C30, 5.0 (−3.4–13.3), *p* = 0.24; at 24 weeks 8.9 (0.9–16.8), *p* = 0.029.	Intention-to-treat analysis in participants with complete data at 24 weeks (24 intervention, 25 controls)

Abbreviations: BMI, body mass index, PACThe, programme of Accompanying women after breast Cancer treatment completion in Thermal resorts; WHI-DM, Women’s Health Initiative—Dietary Modification; LEACH, Leadership and Coaching for Health program; LISA, Lifestyle Intervention in Adjuvant Treatment of Early Breast Cancer Study; HADS, Hospital Anxiety and Depression scale; EORTC QLQ-C30, European Organization for Research and Treatment of Cancer Quality of Life Questionnaire; SF36, Short Form Health Survey; Physical component scale (PCS) and Mental Component Scale (MCS); FACT-B, Function After Cancer Therapy-Breast; FACT-C, Function After Cancer Therapy-Colorectal; FACT-G, Function After Cancer Therapy- excluding the colorectal cancer-specific items; BCPT, Breast Cancer Prevention Trial; BFI, Brief fatigue inventory; PSQI, Pittsburgh sleep quality index; PTGI, post-traumatic growth inventory; PA, physical activity; mo, months; FRD, fatigue reduction diet; APANE, adapted physical activity and nutritional education.

## Data Availability

The data used in this review come from publised articles, all of the identified in the References. The data used in the meta-analysis have been provided in the review’s tables.

## Appendix A

**Table A1:** Risk of bias assessment according to the Newcastle-Ottawa Scale (NOS) for cohort studies.

	Study Reference	Selection (0–4)	Comparability (0–2)	Outcome (0–3)	Total Score (0–9)
Several cancers					
	Inoue-Choi, 2013 *	4	2	3	9
	Karavasiloglou, 2019	4	2	2	8
Breast cancer					
	Kim, 2011	4	2	2	8
	George, 2011	4	1	2	7
	Vrieling, 2013	4	1	2	7
	Inoue-Choi, 2013	4	2	3	9
	Izano, 2013	4	2	2	8
	George, 2014	4	2	2	8
	McCullough, 2016	4	2	2	8
	Jang, 2018	3	1	1	5
	Sun, 2018	4	2	3	9
	Zheng, 2018	4	2	3	9
	Karavasiloglou, 2019	4	2	2	8
	Wang, 2020	4	2	3	9
	Wang, 2021	4	2	2	8
Colorectal cancer					
	Inoue-Choi, 2013	4	2	3	9
	Zhu, 2013	4	2	2	8
	Pelser, 2014	4	2	2	8
	Fung, 2014	4	2	3	9
	Romaguera, 2015	4	2	1	7
	Jacobs, 2016	4	2	2	8
	Yuan, 2017	4	2	2	8
	Ratjen, 2017	4	2	2	8
	Sharma, 2018	4	2	2	8
	Zheng, 2020	4	2	2	8
	Tabung, 2020	4	2	2	8
Prostate cancer					
	Kenfied, 2014	4	2	2	8
	Yang M (1), 2015	4	2	3	9
	Zucchetto, 2016	4	1	2	7
Head and Neck cancer					
	Arthur, 2013	4	1	2	7
	Crowder, 2019	4	1	1	6
Ovarian cancer					
	Thomson, 2014	4	1	1	6
	Hansen, 2020	4	1	2	7
Bladder cancer					
	Westhoff, 2018	4	1	2	7
Multiple myeloma (MM)					
	Lee, 2020	4	1	3	8

Each item included the following subcategories: Selection (0–4 points): Representativeness of the exposed cohort, Selection of the non-exposed cohort, Ascertainment of exposure, Demonstration that outcome of interest was not present at start of study; Comparability (0–2 points): Comparability of cohorts on the basis of the design or analysis; Outcome (0–3): Assessment of outcome, Was follow-up long enough for outcomes to occur, Adequacy of follow-up of cohorts. * The paper by Inoue-Choi 2013 (Ref. [36]) is listed twice since it was considered as two different cohort studies (or two different analyses within the same cohort): one of them analyzed a group of survivors of a group of several cancers, the second one was restricted to breast cancer survivors. Since some items owing to selection and comparability were dealt with differently in each analysis they were considered as two independent studies.

**Table A2:** Summary of cohort study data used for meta-analysis calculation.

		HR (95% CI)					
Study Reference, Cohort	Diet Quality Index	Q1	Q2	Q3	Q4	Q5	10-Unit Increase
Kim, 2011	AHEI						
NHS	-						
	Overall mortality	1.00	0.82 (0.61–1.10)	0.83 (0.62–1.12)	0.98 (0.73–1.32)	0.85 (0.63–1.17)	-
	BC-specific mortality	1.00	1.06 (0.68–1.66)	1.12 (0.72–1.74)	1.28 (0.83–1.98)	1.53 (0.98–2.39)	-
George, 2011	HEI-2005						
HEAL	Mean	50.10	62.90	70.80	79.00	-	
	Overall mortality	1.00	0.39 (0.18–0.85)	0.85 (0.43–1.71)	0.40 (0.17–0.94)	-	0.80 (0.60–1.05)
	BC-specific mortality	1.00	0.65 (0.23–1.86)	0.70 (0.24–2.06)	0.12 (0.02–0.99)	-	0.53 (0.28–0.99)
George 2014	HEI-2005						
WHI	Range (Midpoint)	34–63 (48.5)	63–71 (67)	71–77 (74)	77–91 (84)	-	
	Overall mortality	1.00	0.93 (0.71–1.22)	0.86 (0.65–1.14)	0.74 (0.55–0.99)	-	0.92 (0.85–1.01)
	BC-specific mortality	1.00	0.99 (0.66–1.50)	0.93 (0.61–1.43)	0.91 (0.60–1.40)	-	0.97 (0.86–1.10)
Wang 2020	HEI-2015						
SBCSS	Range (Midpoint)	38.0–58.7 (48.35)	58.7–61.9 (60.3)	61.9–65.8 (63.85)	65.8–78.5 (72.15)	-	
	Overall mortality	1.00	1.08 (0.82–1.42)	1.01 (0.76–1.36)	0.79 (0.57–1.10)	-	0.91 (0.79–1.05)
	BC-specific mortality	1.00	1.10 (0.79–1.53)	0.91 (0.63–1.31)	0.86 (0.58–1.27)	-	0.93 (0.79–1.11)

NHS, Nurses’ Health Study; HEAL, Health, Eating, Activity, and Lifestyle Study; WHI, Women’s Health Initiative; SBCSS, Shanghai Breast Cancer Survival Study; AHEI, Alternative Healthy Eating Index; HEI, healthy eating index; BC, breast cancer; HR, hazard ratio; CI, confidence interval; Q1, first quartile or quintile; Q2, second quartile or quintile; Q3, third quartile or quintile; Q4, fourth quartile or quintile; Q5, fifth quintile.

## References

[1] Ligibel J. (2012). Lifestyle Factors in Cancer Survivorship. J. Clin. Oncol. Off. J. Am. Soc. Clin. Oncol..

[2] Ferlay J., Ervik M., Lam F., Colombet M., Mery L., Piñeros M. (2020). Global Cancer Observatory: Cancer Today.

[3] Allemani C., Weir H.K., Carreira H., Harewood R., Spika D., Wang X.-S., Bannon F., Ahn J.V., Johnson C.J., Bonaventure A. (2015). Global Surveillance of Cancer Survival 1995–2009: Analysis of Individual Data for 25,676,887 Patients from 279 Population-Based Registries in 67 Countries (CONCORD-2). Lancet Lond. Engl..

[4] WCRF/AICR World Cancer Research Fund/American Institute for Cancer Research (2018). Continuous Update Project Expert Report 2018. Survivors of Breast Cancer and Other Cancers. https://www.wcrf.org/wp-content/uploads/2021/02/Cancer-Survivors.pdf.

[5] Schwedhelm C., Boeing H., Hoffmann G., Aleksandrova K., Schwingshackl L. (2016). Effect of Diet on Mortality and Cancer Recurrence among Cancer Survivors: A Systematic Review and Meta-Analysis of Cohort Studies. Nutr. Rev..

[6] Jochems S.H.J., Van Osch F.H.M., Bryan R.T., Wesselius A., van Schooten F.J., Cheng K.K., Zeegers M.P. (2018). Impact of Dietary Patterns and the Main Food Groups on Mortality and Recurrence in Cancer Survivors: A Systematic Review of Current Epidemiological Literature. BMJ Open.

[7] Rinninella E., Mele M.C., Cintoni M., Raoul P., Ianiro G., Salerno L., Pozzo C., Bria E., Muscaritoli M., Molfino A. (2020). The Facts about Food after Cancer Diagnosis: A Systematic Review of Prospective Cohort Studies. Nutrients.

[8] Keaver L., Houlihan C., O’Callaghan N., LaVertu A.E., Ding X., Zhang F.F. (2021). Evidence-Based Nutrition Guidelines for Cancer Survivors in Europe: A Call for Action. Eur. J. Clin. Nutr..

[9] WCRF/AICR World Cancer Research Fund/American Institute for Cancer Research (2018). Diet, Nutrition, Physical Activity and Cancer: A Global Perspective. Continuous Update Project Expert Report 2018. https://www.wcrf.org/wp-content/uploads/2021/02/Summary-of-Third-Expert-Report-2018.pdf.

[10] Steck S.E., Murphy E.A. (2020). Dietary Patterns and Cancer Risk. Nat. Rev. Cancer.

[11] Chlebowski R.T., Blackburn G.L., Thomson C.A., Nixon D.W., Shapiro A., Hoy M.K., Goodman M.T., Giuliano A.E., Karanja N., McAndrew P. (2006). Dietary Fat Reduction and Breast Cancer Outcome: Interim Efficacy Results from the Women’s Intervention Nutrition Study. J. Natl. Cancer Inst..

[12] Pierce J.P., Natarajan L., Caan B.J., Parker B.A., Greenberg E.R., Flatt S.W., Rock C.L., Kealey S., Al-Delaimy W.K., Bardwell W.A. (2007). Influence of a Diet Very High in Vegetables, Fruit, and Fiber and Low in Fat on Prognosis Following Treatment for Breast Cancer: The Women’s Healthy Eating and Living (WHEL) Randomized Trial. JAMA.

[13] Nelson N. (2008). Dietary Intervention Trial Reports No Effect on Survival after Breast Cancer. J. Natl. Cancer Inst..

[14] Bicego D., Brown K., Ruddick M., Storey D., Wong C., Harris S.R. (2009). Effects of Exercise on Quality of Life in Women Living with Breast Cancer: A Systematic Review. Breast J..

[15] Fong D.Y.T., Ho J.W.C., Hui B.P.H., Lee A.M., Macfarlane D.J., Leung S.S.K., Cerin E., Chan W.Y.Y., Leung I.P.F., Lam S.H.S. (2012). Physical Activity for Cancer Survivors: Meta-Analysis of Randomised Controlled Trials. BMJ.

[16] Liberati A., Altman D.G., Tetzlaff J., Mulrow C., Gøtzsche P.C., Ioannidis J.P.A., Clarke M., Devereaux P.J., Kleijnen J., Moher D. (2009). The PRISMA Statement for Reporting Systematic Reviews and Meta-Analyses of Studies That Evaluate Health Care Interventions: Explanation and Elaboration. J. Clin. Epidemiol..

[17] Wells G., Shea B., O’Connell D., Peterson J., Welch V., Losos M. The Newcastle-Ottawa Scale (NOS) for Assessing the Quality If Nonrandomized Studies in Meta-Analyses. http://www.ohri.ca/programs/clinical_epidemiology/oxford.asp.

[18] Kim E.H.J., Willett W.C., Fung T., Rosner B., Holmes M.D. (2011). Diet Quality Indices and Postmenopausal Breast Cancer Survival. Nutr. Cancer.

[19] George S.M., Irwin M.L., Smith A.W., Neuhouser M.L., Reedy J., McTiernan A., Alfano C.M., Bernstein L., Ulrich C.M., Baumgartner K.B. (2011). Postdiagnosis Diet Quality, the Combination of Diet Quality and Recreational Physical Activity, and Prognosis after Early-Stage Breast Cancer. Cancer Causes Control CCC.

[20] George S.M., Ballard-Barbash R., Shikany J.M., Caan B.J., Freudenheim J.L., Kroenke C.H., Vitolins M.Z., Beresford S.A., Neuhouser M.L. (2014). Better Postdiagnosis Diet Quality Is Associated with Reduced Risk of Death among Postmenopausal Women with Invasive Breast Cancer in the Women’s Health Initiative. Cancer Epidemiol. Biomark. Prev. Publ. Am. Assoc. Cancer Res. Cosponsored Am. Soc. Prev. Oncol..

[21] Wang F., Cai H., Gu K., Shi L., Yu D., Zhang M., Zheng W., Zheng Y., Bao P., Shu X.-O. (2020). Adherence to Dietary Recommendations among Long-Term Breast Cancer Survivors and Cancer Outcome Associations. Cancer Epidemiol. Biomark. Prev. Publ. Am. Assoc. Cancer Res. Cosponsored Am. Soc. Prev. Oncol..

[22] Hartemink N., Boshuizen H.C., Nagelkerke N.J.D., Jacobs M.A.M., van Houwelingen H.C. (2006). Combining Risk Estimates from Observational Studies with Different Exposure Cutpoints: A Meta-Analysis on Body Mass Index and Diabetes Type 2. Am. J. Epidemiol..

[23] Deeks J.J., Higgins J.P., Altman D.G. (2008). Analysing Data and Undertaking Meta-Analyses. Cochrane Handbook for Systematic Reviews of Interventions.

[24] Veroniki A.A., Jackson D., Viechtbauer W., Bender R., Bowden J., Knapp G., Kuss O., Higgins J.P.T., Langan D., Salanti G. (2016). Methods to Estimate the Between-Study Variance and Its Uncertainty in Meta-Analysis. Res. Synth. Methods.

[25] Higgins J.P.T., Thompson S.G., Deeks J.J., Altman D.G. (2003). Measuring Inconsistency in Meta-Analyses. BMJ.

[26] Nagashima K., Noma H., Furukawa T.A. (2019). Prediction Intervals for Random-Effects Meta-Analysis: A Confidence Distribution Approach. Stat. Methods Med. Res..

[27] Vrieling A., Buck K., Seibold P., Heinz J., Obi N., Flesch-Janys D., Chang-Claude J. (2013). Dietary Patterns and Survival in German Postmenopausal Breast Cancer Survivors. Br. J. Cancer.

[28] Izano M.A., Fung T.T., Chiuve S.S., Hu F.B., Holmes M.D. (2013). Are Diet Quality Scores after Breast Cancer Diagnosis Associated with Improved Breast Cancer Survival?. Nutr. Cancer.

[29] Jang H., Chung M.S., Kang S.S., Park Y. (2018). Association between the Dietary Inflammatory Index and Risk for Cancer Recurrence and Mortality among Patients with Breast Cancer. Nutrients.

[30] Al-Ibrahim A.A., Jackson R.T. (2019). Healthy Eating Index versus Alternate Healthy Index in Relation to Diabetes Status and Health Markers in U.S. Adults: NHANES 2007–2010. Nutr. J..

[31] Karavasiloglou N., Pestoni G., Faeh D., Rohrmann S. (2019). Post-Diagnostic Diet Quality and Mortality in Females with Self-Reported History of Breast or Gynecological Cancers: Results from the Third National Health and Nutrition Examination Survey (NHANES III). Nutrients.

[32] Sun Y., Bao W., Liu B., Caan B.J., Lane D.S., Millen A.E., Simon M.S., Thomson C.A., Tinker L.F., Van Horn L.V. (2018). Changes in Overall Diet Quality in Relation to Survival in Postmenopausal Women with Breast Cancer: Results from the Women’s Health Initiative. J. Acad. Nutr. Diet..

[33] Zheng J., Tabung F.K., Zhang J., Liese A.D., Shivappa N., Ockene J.K., Caan B., Kroenke C.H., Hébert J.R., Steck S.E. (2018). Association between Post-Cancer Diagnosis Dietary Inflammatory Potential and Mortality among Invasive Breast Cancer Survivors in the Women’s Health Initiative. Cancer Epidemiol. Biomark. Prev. Publ. Am. Assoc. Cancer Res. Cosponsored Am. Soc. Prev. Oncol..

[34] Wang T., Farvid M.S., Kang J.H., Holmes M.D., Rosner B.A., Tamimi R.M., Willett W.C., Eliassen A.H. (2021). Diabetes Risk Reduction Diet and Survival After Breast Cancer Diagnosis. Cancer Res..

[35] McCullough M.L., Gapstur S.M., Shah R., Campbell P.T., Wang Y., Doyle C., Gaudet M.M. (2016). Pre- and Postdiagnostic Diet in Relation to Mortality among Breast Cancer Survivors in the CPS-II Nutrition Cohort. Cancer Causes Control CCC.

[36] Inoue-Choi M., Robien K., Lazovich D. (2013). Adherence to the WCRF/AICR Guidelines for Cancer Prevention Is Associated with Lower Mortality among Older Female Cancer Survivors. Cancer Epidemiol. Biomark. Prev. Publ. Am. Assoc. Cancer Res. Cosponsored Am. Soc. Prev. Oncol..

[37] Chiuve S.E., Fung T.T., Rimm E.B., Hu F.B., McCullough M.L., Wang M., Stampfer M.J., Willett W.C. (2012). Alternative Dietary Indices Both Strongly Predict Risk of Chronic Disease. J. Nutr..

[38] Spineli L.M., Pandis N. (2020). Prediction Interval in Random-Effects Meta-Analysis. Am. J. Orthod. Dentofacial Orthop..

[39] Zhu Y., Wu H., Wang P.P., Savas S., Woodrow J., Wish T., Jin R., Green R., Woods M., Roebothan B. (2013). Dietary Patterns and Colorectal Cancer Recurrence and Survival: A Cohort Study. BMJ Open.

[40] Sharma I., Roebothan B., Zhu Y., Woodrow J., Parfrey P.S., Mclaughlin J.R., Wang P.P. (2018). Hypothesis and Data-Driven Dietary Patterns and Colorectal Cancer Survival: Findings from Newfoundland and Labrador Colorectal Cancer Cohort. Nutr. J..

[41] Fung T.T., Kashambwa R., Sato K., Chiuve S.E., Fuchs C.S., Wu K., Giovannucci E., Ogino S., Hu F.B., Meyerhardt J.A. (2014). Post Diagnosis Diet Quality and Colorectal Cancer Survival in Women. PLoS ONE.

[42] Jacobs S., Harmon B.E., Ollberding N.J., Wilkens L.R., Monroe K.R., Kolonel L.N., Le Marchand L., Boushey C.J., Maskarinec G. (2016). Among 4 Diet Quality Indexes, Only the Alternate Mediterranean Diet Score Is Associated with Better Colorectal Cancer Survival and Only in African American Women in the Multiethnic Cohort. J. Nutr..

[43] Ratjen I., Schafmayer C., di Giuseppe R., Waniek S., Plachta-Danielzik S., Koch M., Nöthlings U., Hampe J., Schlesinger S., Lieb W. (2017). Postdiagnostic Mediterranean and Healthy Nordic Dietary Patterns Are Inversely Associated with All-Cause Mortality in Long-Term Colorectal Cancer Survivors. J. Nutr..

[44] Pelser C., Arem H., Pfeiffer R.M., Elena J.W., Alfano C.M., Hollenbeck A.R., Park Y. (2014). Prediagnostic Lifestyle Factors and Survival after Colon and Rectal Cancer Diagnosis in the National Institutes of Health (NIH)-AARP Diet and Health Study. Cancer.

[45] Zheng J., Tabung F.K., Zhang J., Murphy E.A., Shivappa N., Ockene J.K., Caan B., Kroenke C.H., Hébert J.R., Steck S.E. (2020). Post-Cancer Diagnosis Dietary Inflammatory Potential Is Associated with Survival among Women Diagnosed with Colorectal Cancer in the Women’s Health Initiative. Eur. J. Nutr..

[46] Tabung F.K., Noonan A., Lee D.H., Song M., Clinton S.K., Spakowicz D., Wu K., Cheng E., Meyerhardt J.A., Fuchs C.S. (2020). Post-Diagnosis Dietary Insulinemic Potential and Survival Outcomes among Colorectal Cancer Patients. BMC Cancer.

[47] Romaguera D., Ward H., Wark P.A., Vergnaud A.-C., Peeters P.H., van Gils C.H., Ferrari P., Fedirko V., Jenab M., Boutron-Ruault M.-C. (2015). Pre-Diagnostic Concordance with the WCRF/AICR Guidelines and Survival in European Colorectal Cancer Patients: A Cohort Study. BMC Med..

[48] Kenfield S.A., DuPre N., Richman E.L., Stampfer M.J., Chan J.M., Giovannucci E.L. (2014). Mediterranean Diet and Prostate Cancer Risk and Mortality in the Health Professionals Follow-up Study. Eur. Urol..

[49] Yang M., Kenfield S.A., Van Blarigan E.L., Batista J.L., Sesso H.D., Ma J., Stampfer M.J., Chavarro J.E. (2015). Dietary Patterns after Prostate Cancer Diagnosis in Relation to Disease-Specific and Total Mortality. Cancer Prev. Res..

[50] Zucchetto A., Gini A., Shivappa N., Hébert J.R., Stocco C., Dal Maso L., Birri S., Serraino D., Polesel J. (2016). Dietary Inflammatory Index and Prostate Cancer Survival. Int. J. Cancer.

[51] Arthur A.E., Peterson K.E., Rozek L.S., Taylor J.M.G., Light E., Chepeha D.B., Hébert J.R., Terrell J.E., Wolf G.T., Duffy S.A. (2013). Pretreatment Dietary Patterns, Weight Status, and Head and Neck Squamous Cell Carcinoma Prognosis. Am. J. Clin. Nutr..

[52] Crowder S.L., Sarma K.P., Mondul A.M., Chen Y.T., Li Z., Pepino M.Y., Zarins K.R., Wolf G.T., Rozek L.S., Arthur A.E. (2019). Pretreatment Dietary Patterns Are Associated with the Presence of Nutrition Impact Symptoms 1 Year after Diagnosis in Patients with Head and Neck Cancer. Cancer Epidemiol. Biomark. Prev. Publ. Am. Assoc. Cancer Res. Cosponsored Am. Soc. Prev. Oncol..

[53] Thomson C.A., Crane T.E., Wertheim B.C., Neuhouser M.L., Li W., Snetselaar L.G., Basen-Engquist K.M., Zhou Y., Irwin M.L. (2014). Diet Quality and Survival after Ovarian Cancer: Results from the Women’s Health Initiative. J. Natl. Cancer Inst..

[54] Hansen J.M., Nagle C.M., Ibiebele T.I., Grant P.T., Obermair A., Friedlander M.L., DeFazio A., Webb P.M. (2020). Ovarian Cancer Prognosis and Lifestyle Study Group A Healthy Lifestyle and Survival among Women with Ovarian Cancer. Int. J. Cancer.

[55] Westhoff E., Wu X., Kiemeney L.A., Lerner S.P., Ye Y., Huang M., Dinney C.P., Vrieling A., Tu H. (2018). Dietary Patterns and Risk of Recurrence and Progression in Non-Muscle-Invasive Bladder Cancer. Int. J. Cancer.

[56] Lee D.H., Fung T.T., Tabung F.K., Marinac C.R., Devore E.E., Rosner B.A., Ghobrial I.M., Colditz G.A., Giovannucci E.L., Birmann B.M. (2020). Prediagnosis Dietary Pattern and Survival in Patients with Multiple Myeloma. Int. J. Cancer.

[57] Zick S.M., Colacino J., Cornellier M., Khabir T., Surnow K., Djuric Z. (2017). Fatigue Reduction Diet in Breast Cancer Survivors: A Pilot Randomized Clinical Trial. Breast Cancer Res. Treat..

[58] Bourke L., Thompson G., Gibson D.J., Daley A., Crank H., Adam I., Shorthouse A., Saxton J. (2011). Pragmatic Lifestyle Intervention in Patients Recovering from Colon Cancer: A Randomized Controlled Pilot Study. Arch. Phys. Med. Rehabil..

[59] Koutoukidis D.A., Beeken R.J., Manchanda R., Burnell M., Ziauddeen N., Michalopoulou M., Knobf M.T., Lanceley A. (2019). Diet, Physical Activity, and Health-Related Outcomes of Endometrial Cancer Survivors in a Behavioral Lifestyle Program: The Diet and Exercise in Uterine Cancer Survivors (DEUS) Parallel Randomized Controlled Pilot Trial. Int. J. Gynecol. Cancer Off. J. Int. Gynecol. Cancer Soc..

[60] Goodwin P.J., Segal R.J., Vallis M., Ligibel J.A., Pond G.R., Robidoux A., Blackburn G.L., Findlay B., Gralow J.R., Mukherjee S. (2014). Randomized Trial of a Telephone-Based Weight Loss Intervention in Postmenopausal Women with Breast Cancer Receiving Letrozole: The LISA Trial. J. Clin. Oncol. Off. J. Am. Soc. Clin. Oncol..

[61] Demark-Wahnefried W., Colditz G.A., Rock C.L., Sedjo R.L., Liu J., Wolin K.Y., Krontiras H., Byers T., Pakiz B., Parker B.A. (2015). Quality of Life Outcomes from the Exercise and Nutrition Enhance Recovery and Good Health for You (ENERGY)-Randomized Weight Loss Trial among Breast Cancer Survivors. Breast Cancer Res. Treat..

[62] Swisher A.K., Abraham J., Bonner D., Gilleland D., Hobbs G., Kurian S., Yanosik M.A., Vona-Davis L. (2015). Exercise and Dietary Advice Intervention for Survivors of Triple-Negative Breast Cancer: Effects on Body Fat, Physical Function, Quality of Life, and Adipokine Profile. Support. Care Cancer Off. J. Multinatl. Assoc. Support. Care Cancer.

[63] Scott E., Daley A.J., Doll H., Woodroofe N., Coleman R.E., Mutrie N., Crank H., Powers H.J., Saxton J.M. (2013). Effects of an Exercise and Hypocaloric Healthy Eating Program on Biomarkers Associated with Long-Term Prognosis after Early-Stage Breast Cancer: A Randomized Controlled Trial. Cancer Causes Control.

[64] Kwiatkowski F., Mouret-Reynier M.-A., Duclos M., Bridon F., Hanh T., Van Praagh-Doreau I., Travade A., Vasson M.-P., Jouvency S., Roques C. (2017). Long-Term Improvement of Breast Cancer Survivors’ Quality of Life by a 2-Week Group Physical and Educational Intervention: 5-Year Update of the “PACThe” Trial. Br. J. Cancer.

[65] Ruiz-Vozmediano J., Löhnchen S., Jurado L., Recio R., Rodríguez-Carrillo A., López M., Mustieles V., Expósito M., Arroyo-Morales M., Fernández M.F. (2020). Influence of a Multidisciplinary Program of Diet, Exercise, and Mindfulness on the Quality of Life of Stage IIA-IIB Breast Cancer Survivors. Integr. Cancer Ther..

[66] Chlebowski R.T., Aragaki A.K., Anderson G.L., Pan K., Neuhouser M.L., Manson J.E., Thomson C.A., Mossavar-Rahmani Y., Lane D.S., Johnson K.C. (2020). Dietary Modification and Breast Cancer Mortality: Long-Term Follow-Up of the Women’s Health Initiative Randomized Trial. J. Clin. Oncol. Off. J. Am. Soc. Clin. Oncol..

[67] Yun Y.H., Kim Y.A., Lee M.K., Sim J.A., Nam B.-H., Kim S., Lee E.S., Noh D.-Y., Lim J.-Y., Kim S. (2017). A Randomized Controlled Trial of Physical Activity, Dietary Habit, and Distress Management with the Leadership and Coaching for Health (LEACH) Program for Disease-Free Cancer Survivors. BMC Cancer.

[68] Ho M., Ho J.W.C., Fong D.Y.T., Lee C.F., Macfarlane D.J., Cerin E., Lee A.M., Leung S., Chan W.Y.Y., Leung I.P.F. (2020). Effects of Dietary and Physical Activity Interventions on Generic and Cancer-Specific Health-Related Quality of Life, Anxiety, and Depression in Colorectal Cancer Survivors: A Randomized Controlled Trial. J. Cancer Surviv..

[69] Bonelli L., Puntoni M., Gatteschi B., Massa P., Missale G., Munizzi F., Turbino L., Villanacci V., De Censi A., Bruzzi P. (2013). Antioxidant Supplement and Long-Term Reduction of Recurrent Adenomas of the Large Bowel. A Double-Blind Randomized Trial. J. Gastroenterol..

[70] Parsons J.K., Zahrieh D., Mohler J.L., Paskett E., Hansel D.E., Kibel A.S., Liu H., Seisler D.K., Natarajan L., White M. (2020). Effect of a Behavioral Intervention to Increase Vegetable Consumption on Cancer Progression Among Men With Early-Stage Prostate Cancer: The MEAL Randomized Clinical Trial. JAMA.

[71] George S.M., Neuhouser M.L., Mayne S.T., Irwin M.L., Albanes D., Gail M.H., Alfano C.M., Bernstein L., McTiernan A., Reedy J. (2010). Postdiagnosis Diet Quality Is Inversely Related to a Biomarker of Inflammation among Breast Cancer Survivors. Cancer Epidemiol. Biomark. Prev. Publ. Am. Assoc. Cancer Res. Cosponsored Am. Soc. Prev. Oncol..

[72] Bouvard V., Loomis D., Guyton K.Z., Grosse Y., Ghissassi F.E., Benbrahim-Tallaa L., Guha N., Mattock H., Straif K. (2015). International Agency for Research on Cancer Monograph Working Group Carcinogenicity of Consumption of Red and Processed Meat. Lancet Oncol..

[73] McMillan D.C. (2013). The Systemic Inflammation-Based Glasgow Prognostic Score: A Decade of Experience in Patients with Cancer. Cancer Treat. Rev..

[74] Agudo A., Cayssials V., Bonet C., Tjønneland A., Overvad K., Boutron-Ruault M.-C., Affret A., Fagherazzi G., Katzke V., Schübel R. (2018). Inflammatory Potential of the Diet and Risk of Gastric Cancer in the European Prospective Investigation into Cancer and Nutrition (EPIC) Study. Am. J. Clin. Nutr..

[75] Buckland G., Travier N., Arribas L., Del Barco S., Pernas S., Zamora E., Bellet M., Cirauqui B., Margelí M., Muñoz M. (2019). Changes in Dietary Intake, Plasma Carotenoids and Erythrocyte Membrane Fatty Acids in Breast Cancer Survivors after a Lifestyle Intervention: Results from a Single-Arm Trial. J. Hum. Nutr. Diet. Off. J. Br. Diet. Assoc..

[76] Guinter M.A., McLain A.C., Merchant A.T., Sandler D.P., Steck S.E. (2018). A Dietary Pattern Based on Estrogen Metabolism Is Associated with Breast Cancer Risk in a Prospective Cohort of Postmenopausal Women. Int. J. Cancer.

[77] Tabung F.K., Wang W., Fung T.T., Hu F.B., Smith-Warner S.A., Chavarro J.E., Fuchs C.S., Willett W.C., Giovannucci E.L. (2016). Development and Validation of Empirical Indices to Assess the Insulinemic Potential of Diet and Lifestyle. Br. J. Nutr..

[78] Ligibel J.A., Alfano C.M., Hershman D., Ballard R.M., Bruinooge S.S., Courneya K.S., Daniels E.C., Demark-Wahnefried W., Frank E.S., Goodwin P.J. (2015). Recommendations for Obesity Clinical Trials in Cancer Survivors: American Society of Clinical Oncology Statement. J. Clin. Oncol..

